# Siglec-7 restores β-cell function and survival and reduces inflammation in pancreatic islets from patients with diabetes

**DOI:** 10.1038/srep45319

**Published:** 2017-04-05

**Authors:** Gitanjali Dharmadhikari, Katharina Stolz, Michael Hauke, Noel G. Morgan, Ajit Varki, Eelco de Koning, Sørge Kelm, Kathrin Maedler

**Affiliations:** 1 Centre for Biomolecular Interactions Bremen, University of Bremen, Germany; 2 Hubrecht Institute-KNAW (Royal Netherlands Academy of Arts and Sciences) and University Medical Center Utrecht, Cancer Genomics Netherlands, Utrecht, The Netherlands; 3 Institute of Biomedical and Clinical Sciences, University of Exeter Medical School, RILD Building, Barrack Road, Exeter EX2 5DW, UK; 4 Glycobiology Research and Training Center and Departments of Medicine and Cellular and Molecular Medicine, University of California at San Diego, La Jolla, CA, USA; 5 Leiden University Medical Center, Department of Medicine, Section of Nephrology and Section of Endocrinology, Leiden, the Netherlands

## Abstract

Chronic inflammation plays a key role in both type 1 and type 2 diabetes. Cytokine and chemokine production within the islets in a diabetic milieu results in β-cell failure and diabetes progression. Identification of targets, which both prevent macrophage activation and infiltration into islets and restore β-cell functionality is essential for effective diabetes therapy. We report that certain Sialic-acid-binding immunoglobulin-like-lectins (siglecs) are expressed in human pancreatic islets in a cell-type specific manner. Siglec-7 was expressed on β-cells and down-regulated in type 1 and type 2 diabetes and in infiltrating activated immune cells. Over-expression of Siglec-7 in diabetic islets reduced cytokines, prevented β-cell dysfunction and apoptosis and reduced recruiting of migrating monocytes. Our data suggest that restoration of human Siglec-7 expression may be a novel therapeutic strategy targeted to both inhibition of immune activation and preservation of β-cell function and survival.

Diabetes mellitus is a syndrome of disordered glucose metabolism, caused by a combination of hereditary and environmental factors, which result in hyperglycemia. The ability of the β-cells to secrete adequate amounts of insulin to maintain normoglycemia depends on their function and mass. In both Type 1 diabetes mellitus (T1D) and Type 2 diabetes mellitus (T2D), the major mechanism leading to decreased β-cell mass is increased β-cell apoptosis^1^. T1D results from an absolute insulin deficiency due to the autoimmune destruction of the insulin producing β-cells^2^,^3^. β-cell destruction occurs through immune mediated processes such as mononuclear cell infiltration in the pancreatic islets and interaction between antigen presenting cells and T-cells, which leads to high local concentrations of inflammatory cytokines, chemokines, reactive oxygen species (ROS) and other inflammatory products, and subsequently to β-cell apoptosis. T2D is strongly associated with obesity and characterized by chronic insulin resistance and a progressive decline in β-cell function and mass^4^. A chronic, low-grade inflammatory state is present in obesity, with adipose tissue macrophage infiltration and pro-inflammatory activity of macrophages^5^. Epidemiological studies suggest that low-grade inflammation precedes and predicts the development of T2D^6^. Cytokines and chemokines are produced and secreted not only by activated infiltrating macrophages, but also by adipocytes and pancreatic β-cells themselves. The chronic elevation of glucose and free fatty acid levels occurring in diabetes triggers a pro-inflammatory response in several tissues such as adipose tissue, muscle, liver, immune cells and also the islets^7^. Pro-inflammatory cytokines can cause insulin resistance^8^, impair β-cell function^9^, and anti-inflammatory mediators may reverse both effects^10^,^11^, implying that inflammation may be directly involved in the pathogenesis of T2D. Hence, activation of the innate immune system and triggering of local as well as systemic inflammation are hallmarks of both T1D and T2D.

Signaling and activation of immune cells is brought about by secreted stimuli as well as via cell-cell interactions. Different cell surface receptors and adhesion molecules play a role in the immune activation. One such family of adhesion and signaling molecules are Sialic acid-binding immunoglobulin-like lectins (siglecs)^12^. Siglecs are I-type lectins, which recognize and interact via immunoglobulin (Ig)-like domains with sialylated glycan residues on the same cell surface (*cis*-interaction) or on neighboring cells, on extracellular matrix proteins or on secreted glycoproteins (*trans*-interactions). On the basis of evolutionary sequence conservation, the siglecs can be divided into two distinct groups: a subgroup that is highly conserved between species (Siglecs-1, -2, -4 and -15) and a fast evolving CD33/Siglec-3-related subgroup (Siglecs-3, -5 to -12 and 14 and 16 in humans)^13^. Most siglecs are differentially expressed on hematopoietic and immune cells. So far, the exceptions are, Siglec-4 (myelin-associated glycoprotein, MAG) on glial cells^14^, Siglec-5 and 14 on human amniotic epithelium^15^, Siglec-6 on the human placental trophoblast^16^, Siglec-11 on ovarian fibroblasts^17^ and Siglec-12 on certain hominid epithelial cells^18^,^19^.

Each siglec recognizes specific sialic acid linkages in the context of the underlying glycan moiety in the glycocalyx, thus hinting towards their unique function^20^. Typically, cytoplasmic motifs of inhibitory siglecs show the presence of one or more immunoreceptor tyrosine-based inhibitory motifs (ITIMs). These ITIMs recruit tyrosine phosphatases and eventually inhibit activation signals transduced from other receptors. Certain other siglecs can recruit the activating DAP-12 adaptor^18^. These signalling processes can contribute to the functioning of the immune system^21^.

Siglec-1 was recently linked to autoimmune diabetes: increased Siglec-1 expression in monocytes correlates with the presence of a type I IFN signature prior to the development of islet autoimmunity^22^.

To look for the presence and function of siglecs in infiltrating immune cells in islets, we investigated expression levels in the human pancreas. Surprisingly, we found the presence of siglecs in non-immune cells. We show cell type specific siglec expression in the human endocrine pancreas. Because of its specific presence on β-cells, we focused our experimentation on Siglec-7. Cloned for the first time in 1999^23^, Siglec-7 is a CD33-related siglec constitutively expressed on all natural killer (NK) cells, monocytes and also on a subset of T cells^24^. Structurally, it is characterized by 3 immunoglobulin-like extracellular domains (one amino-terminal V-set type and two C2-set type), a trans-membrane region and a cytoplasmic tail containing two tyrosine residues located in immunoreceptor tyrosine-based inhibitory motifs. Siglec-7 acts as an inhibitory receptor in human NK cells after engagement by antibodies^23^ or binding of sialic acid-containing ligands^25^. Upon phosphorylation, it can recruit the SH2 domain-bearing protein tyrosine phosphatase (PTP) SHP-1^23^. Anti-Siglec-7 antibodies inhibit the proliferation of myeloid cells^26^. Also, Siglec-7 inhibits FcRI-mediated serotonin release from RBL cells following crosslinking. The ITIMs are essential for this inhibitory function, and facilitate tyrosine phosphorylation and recruitment of SHP-1 and SHP-2 phosphatases^27^. Siglec-7 is also expressed on a minor subset of T-cells and may negatively regulate T-cell receptor (TCR) signaling^28^. Thus, Siglec-7 can be considered as an inhibitory receptor, participating in the regulation of cell function and survival.

The present study identifies an unexpected role of siglecs in the manifestation and progression of T1D and T2D. We investigated whether inhibitory signals by Siglec-7 can restore β-cell survival and function in a diabetic milieu and whether Siglec-7 expression can influence immune cell migration *in vitro*.

## Results

### Siglecs are differentially expressed in human pancreatic islets

**Figure 1:**
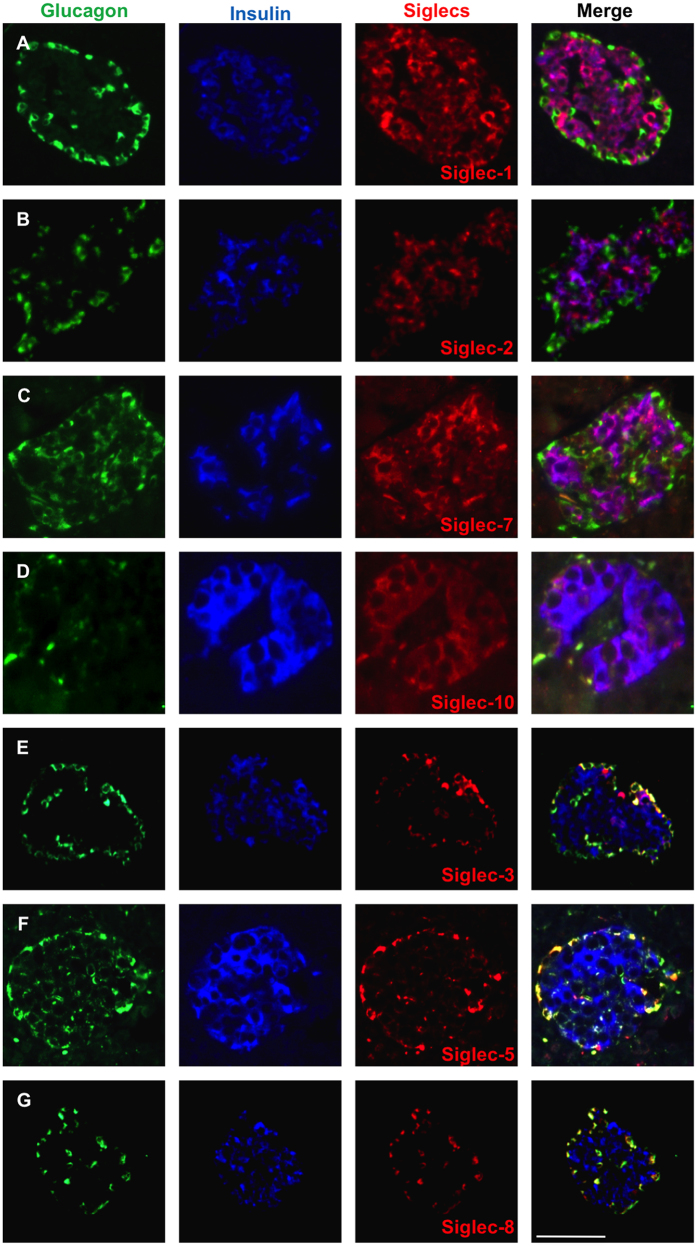
Siglecs are differentially expressed in the human Islets of Langerhans. Triple immunostaining for insulin (blue), glucagon (green) and siglecs (red) was carried out on human pancreatic sections obtained at autopsy from non-diabetic individuals. (**A**) Siglecs-1, (**B**) -2, (**C**) -7and (**D**) -10 were expressed in β-cells. (**E**) Siglecs-3, (**F**) -5, (**G**) -8 were expressed solely in α-cells. Representative analyses from 5 pancreases from age and weight-matched patients with T2D and 5 controls are shown. Bar is 100 μm.

Siglecs are classically expressed in the cells of the hematopoietic system and regulate the inflammatory cell response. Since many pattern recognition receptors and cytokine receptors are highly expressed on β-cells, we assessed the expression levels of the 10 human siglecs in the human pancreas using well characterized polyclonal monospecific antibodies (reviewed in ref. 29). Immunofluorescent labeling in combination with insulin and glucagon staining revealed the presence of siglecs predominantly in the endocrine pancreas. The evolutionarily conserved Siglec-1 (Sialoadhesin) and Siglec-2 (CD22); as well as Siglec-7 and -10 were expressed exclusively in insulin-producing β-cells (Fig. 1A–D) and Siglec-3, -5, and -8 were expressed solely by the glucagon-producing α-cells (Fig. 1E–G). In contrast, Siglec-4, -6 and -9 were not expressed in pancreatic islets (not shown). The specific cellular localization of Siglec-7 was confirmed by carrying out confocal laser scanning microscopy (Suppl. Fig. 1A).

### Siglec-7 and -3 are reciprocally regulated in diabetes

**Figure 2:**
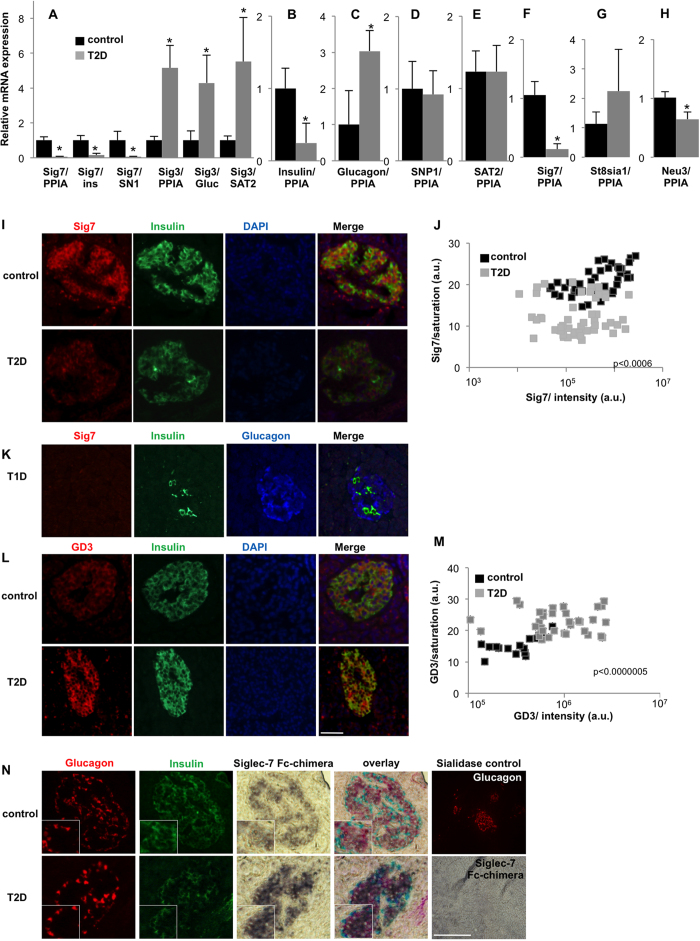
Siglec-7 and -3 are reciprocally regulated in type 2 diabetes. Semi quantitative real time PCR analysis was performed on cDNAs obtained from autopsy pancreases from non-diabetic (n = 9) and individuals with T2D (n = 5), the latter all with documented fasting plasma glucose >150 mg/dl. (**A**) Siglec-7 expression was normalized on cyclophilin (PPIA), insulin (ins) and SN1; whereas Siglec-3 expression was normalized on cyclophilin, glucagon (gluc) and SAT2. (**B–E**) Insulin, Glucagon, SN1 and SAT2 were normalized on cyclophilin. (**F–H**) Real time PCR analysis of freshly isolated islets of patients with T2D (n = 5) were compared to that of non-diabetic individuals (n = 3) of (**F**) Siglec-7, (**G**) St8Sia1 and (**H**) Neu3 (**I,J**). Immunohistochemical analysis was carried out on human pancreatic sections obtained at autopsy of non-diabetic controls and patients with T2D for (**I,J**) Siglec-7, (**L**) GD3. (**J,M**) staining saturation and intensity were quantified using Photoshop; each data point represents saturation and intensity of the protein signal of islets from an average of 42 islets from 3 donors, respectively. (**K**) Analysis of Siglec-7 and insulin in the pancreas of a patient with T1D with remaining insulin^+^β-cells. (**N**) Bright field staining using Siglec-7 Fc-chimeras along with glucagon (red) and insulin (green); along with control slides treated with sialidase treatment. Bar is 100 μm.

In order to understand the role of siglec expression in the endocrine pancreas, we investigated whether their expression is altered in diabetes, semi-quantitative real time PCR analysis was performed on *SIGLEC* cDNAs obtained from autopsy pancreases from non-diabetic patients and patients with T2D. In addition to housekeeping genes, expression levels of *SIGLEC*s were normalized to cell specific markers of β- and α-cells i.e. insulin and glucagon, to account for the changes in their mass in individuals with diabetes. In addition, pancreatic *SIGLEC* expression was normalized to the β- and α-cell specific glutamate receptors SN1 and SAT2, whose expression is unaltered in diabetes^30^. Siglec-7 expression on β-cells was drastically decreased in individuals with T2D when normalized to expression levels of cyclophilin (PPIA), insulin and SN1 (Fig. 2A; reduced by 94%, 85%, 94% respectively vs. control). Also, Siglec-10 was significantly down-regulated in T2D as compared to cyclophilin (PPIA) and SN1 and showed a similar tendency when normalized to insulin (Supp. Fig. 1C). On the other hand, the α-cell specific Siglec-3 showed a substantial increase in diabetes upon normalization against cyclophilin (PPIA), glucagon or SAT2 (Fig. 2A; induced to 5.15-, 4.29-, 5.52-fold, respectively in individuals with T2D, vs. non-diabetic controls). A decrease in insulin mRNA was confirmed in T2D (Fig. 2B), while glucagon mRNA showed an increase in T2D (Fig. 2C) and β- and α-cell specific SN1 and SAT2 remained unchanged in T2D (Fig. 2D,E).

The down-regulation of β-cell *SIGLEC* mRNAs was confirmed in freshly isolated human islets from organ donors with T2D and controls. *SIGLEC7* showed 87% reduction vs. non-diabetic control islets (Fig. 2F) and *SIGLEC10* showed a similar decrease (Suppl. Fig. 1D). Because of the β-cell specific expression and significant regulation in diabetes, we focused our subsequent work on the presence and implication of Siglec-7 in the progression of diabetes.

Siglecs bind to different linkages of the terminal sialic acid to its underlying glycan with varying affinities^31^. Siglec-7 has a binding preference for α2,8-linked disialic acid, which leads to downstream signaling via its cytoplasmic inhibitory motifs^32^. In contrast to Siglec-7, the sialyl-transferase responsible for α2,8 linkage formation, St8Sia1 showed a tendency for up-regulation in the islets from patients with T2D (Fig. 2G), suggestive of a compensatory mechanism and in confirmation of a very recent study which shows St8Sia1 protein upregulation in T2D islets^33^.

The membrane-associated sialic acid-cleaving enzyme sialidase Neu3 (Fig. 2H), which may unmask Siglec-7 residues and thus induce Siglec-7 mediated inhibition of cell death^25^, was significantly down-regulated in islets isolated from patients with T2D, which is a further potential deleterious mechanism in the inflammation-initiation cascade.

The expression of Siglec-7 in human islets was confirmed by flow cytometric analysis of dispersed islet cells (Suppl. Fig. 2A). The loss of Siglec-7 expression was also quantified in β-cells of autopsy human pancreas sections by immunofluorescent labeling and showed a 60% and 63% decrease in intensity and saturation respectively in patients with T2D (all with documented fasting plasma glucose >150 mg/dl) vs. non-diabetic controls (Fig. 2I,J). Such loss in Siglec-7 also occurred in the residual insulin-containing islets of patients with T1D chosen from among a cohort studied previously^34^. Almost no Siglec-7 expression could be seen in any of the insulin-containing islets (Fig. 2K, Suppl. Fig. 1A), while α-cells continued to express Siglec-3 (Suppl. Fig. 1B).

The disialoganglioside GD3 is one of the endogenous ligands for Siglec-7 which displays α2,8-linked disialic acids^35^. High levels of GD3 reverse the protective actions of Siglec-7 on cell survival^25^. Constitutive expression of GD3 was detected in the β-cells of autopsy human pancreas sections by immunofluorescent labeling. As compared to non-diabetic individuals, GD3 was more strongly expressed in patients with T2D. Quantification of the staining showed a 1.50-fold and 3.27-fold increase in intensity and saturation respectively in non-diabetic controls vs. patients with T2D (Fig. 2L,M).

We further confirmed the reciprocal regulation of Siglec-7 and its ligands in T2D by another biochemical approach. Chimeric proteins consisting of the IgG like V-set domain attached to an Fc-region were constructed, expressed and purified from the CHO-Lec1 cell line^36^. They were used as probes to detect the presence of Siglec-7 binding partners. As a negative control, slides treated with sialidase were probed, in which there was no detection of ligands. Bright field staining of the chimeras revealed the presence of Siglec-7 ligands in both α- and β-cells (Fig. 2N), which was increased in pancreatic sections of patients with T2D. These findings hint towards a disruption of Siglec-7 engagement in islets from patients with T2D, with the cells attempting to counteract this by up-regulating relevant ligands.

### Siglec-7 over-expression improves β-cell survival and function

In order to understand the physiological impact of decreased Siglec-7 expression in diabetes, we used an *in vitro* model of human islets exposed to a diabetogenic milieu of elevated glucose (22.2–33.3 mM; HG) and free fatty acid (0.5 mM palmitate; Pal) or the cytokine mixture of 2 ng/ml IL-1β and 1,000 IU/ml IFNγ (IL/IF). Re-expression of Siglec-7 was then achieved to examine whether Siglec-7 restores β-cell function and survival under diabetogenic conditions. Siglec-7 was overexpressed by liposome-mediated transfection. Cell surface expression of Siglec-7 in islets and antibody specificity was confirmed by flow cytometric analysis of dispersed islets and HEK293 cells overexpressing Siglec-7 (Suppl. Fig. 2A,B), by immunocytochemistry of Siglec-7 overexpressing human islet sections (Supp. Fig. 2C,D) and by western blot analysis of Siglec-7 overexpressing and depleted human islets, in human PBMCs and in Siglec-7 overexpressing CHO cells^24^ (Suppl. Fig. 2E).

**Figure 3:**
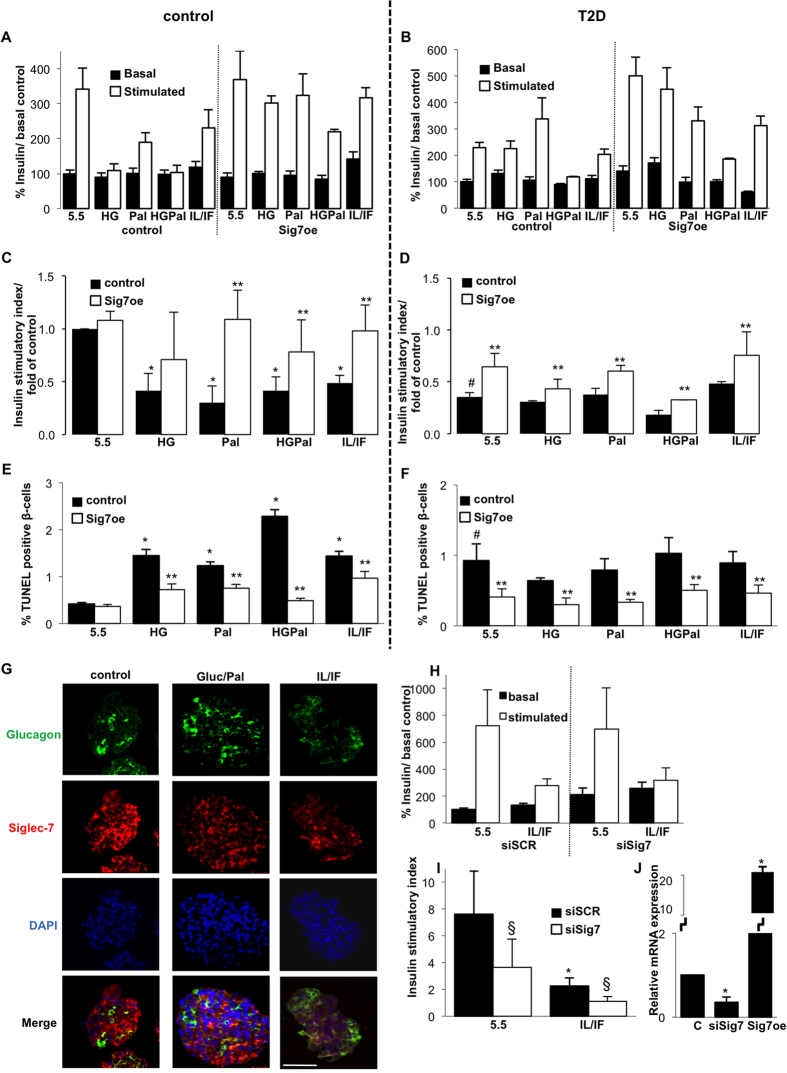
Siglec-7 over-expression improves β-cell survival and function. Freshly isolated human islets of non-diabetic individuals as well as from patients with T2D were cultured on extracellular matrix-coated dishes and exposed to elevated glucose concentrations (22.2 or 33.3 mM; HG-both had the same effect and thus results were combined) with or without palmitate (HGPal), palmitate alone (Pal) or the cytokine mixture IL-1β (2 ng/ml) and IFNγ (1,000 U/ml) (IL/IF) for 72 h with or without over-expression by lipofectamine-mediated Siglec-7 plasmid transfection. Glucose stimulated insulin secretion assays were performed after the 72 h culture period. (**A,B**) Basal (2.8 mM) and glucose stimulated (16.7 mM) insulin secretion was expressed as percent change of control condition basal insulin levels. (**C,D**) Stimulatory index denotes the amount of glucose stimulated (16.7 mM glucose) divided by the amount of basal insulin secretion. Fold changes in stimulatory indices of treated islets were plotted, compared to stimulatory index of control islets. (**E,F**) Apoptosis was analyzed by the TUNEL assay in dishes. Islets were triple-stained for insulin and counterstained for DAPI (not shown). Results are means ± SE of the percentage of TUNEL-positive β-cells. The average number of β-cells counted was 8124 for each treatment group in 3–4 separate experiments from 3 separate dishes per treatment from 3–4 different organ donors. (**G**) Isolated human islets were treated with 22.2 mM glucose and 0.5 mM palmitate; or the cytokine mixture IL/IF, followed by immunohistochemical analysis of paraffin-embedded islet sections. Representative images show glucagon (green), Siglec-7 (red) and DAPI (blue). (**H,I**) Human islets were transfected with 100 nM siRNA to Siglec-7 or scrambled control (siSCR) and treated with the cytokine mixture IL/IF for 72 h. (**H**) Basal (2.8 mM) and glucose stimulated (16.7 mM) insulin secretion and (**I**) stimulatory index were analyzed as above. (**J**) Siglec mRNA expression in islets transfected with siRNA to Siglec-7 or Siglec-7 plasmid DNA. *p < 0.05 to 5.5 mM glucose non-treated control islets, **p < 0.05 Siglec-7 vs. LacZ transfected islets under same diabetic stimuli, ^#^p < 0.05 to 5.5 mM glucose treated LacZ transfected non-diabetic control islets. ^§^p < 0.05 to scramble siRNA treated islets under the same treatment. Data are shown as mean ± SE. Experiments were performed in triplicates, respectively from at least 3–5 independent experiments per condition from 3–5 human islet donors. Bar is 100 μm.

β-cell function (Fig. 3A–D) and survival (Fig. 3E,F) were impaired by all diabetogenic culture conditions (Fig. 3A,C,E) as well as in islets isolated from patients with T2D (Fig. 3B,D,F). Importantly, Siglec-7 over-expression improved β-cell function and survival as measured by TUNEL- and by monitoring the number of insulin positive cells under all diabetogenic conditions (Fig. 3A–F) in non-diabetic islets as well as in islets isolated from T2D organ donors. In the case of islets from donors with T2D, Siglec-7 over-expression completely normalized β-cell function and survival (Fig. 3B,D,F). Islets from patients with T2D showed a 60% loss in glucose stimulated insulin secretion and a 2.2-fold increase in apoptosis, compared to islets isolated from non-diabetic controls (p < 0.05, Fig. 3D,F). Culture of islets from patients with T2D in a diabetic milieu did not further impair β-cell function and survival, but Siglec-7 overexpression restored GSIS and survival under all conditions.

In line with our observations in the pancreases from patients with diabetes, exposure of islets to diabetogenic conditions *in vitro* led to a loss of Siglec-7 expression (Fig. 3G), in particular, treatment of islets with a mixture of 22.2 mM glucose/0.5 mM palmitate as well as with the cytokines IL-1β/IFNγ.

To establish whether a loss in Siglec-7 alone is sufficient to impair β-cell function and survival, we downregulated Siglec-7 in human islets by the use of specific siRNA to Siglec-7. The loss of Siglec-7 alone (65% depletion of Siglec-7 mRNA, analysed by RT-PCR; Fig. 3J, protein depletion was confirmed by Western blotting, Suppl. Fig. 2E) impaired β-cell function *in vitro* (Fig. 3H,I; 60% reduction in GSIS, as compared to scramble transfected control islets). In addition, down-regulation of Siglec-7 significantly potentiated the deleterious effects of cytokines on GSIS in human islets. This was due to an elevated basal insulin secretion and suggests insulin release at low glucose concentration from apoptotic cells induced by Siglec-7 depletion in culture.

### Siglec-7 over-expression inhibits NF-κB activation and cytokine production

**Figure 4:**
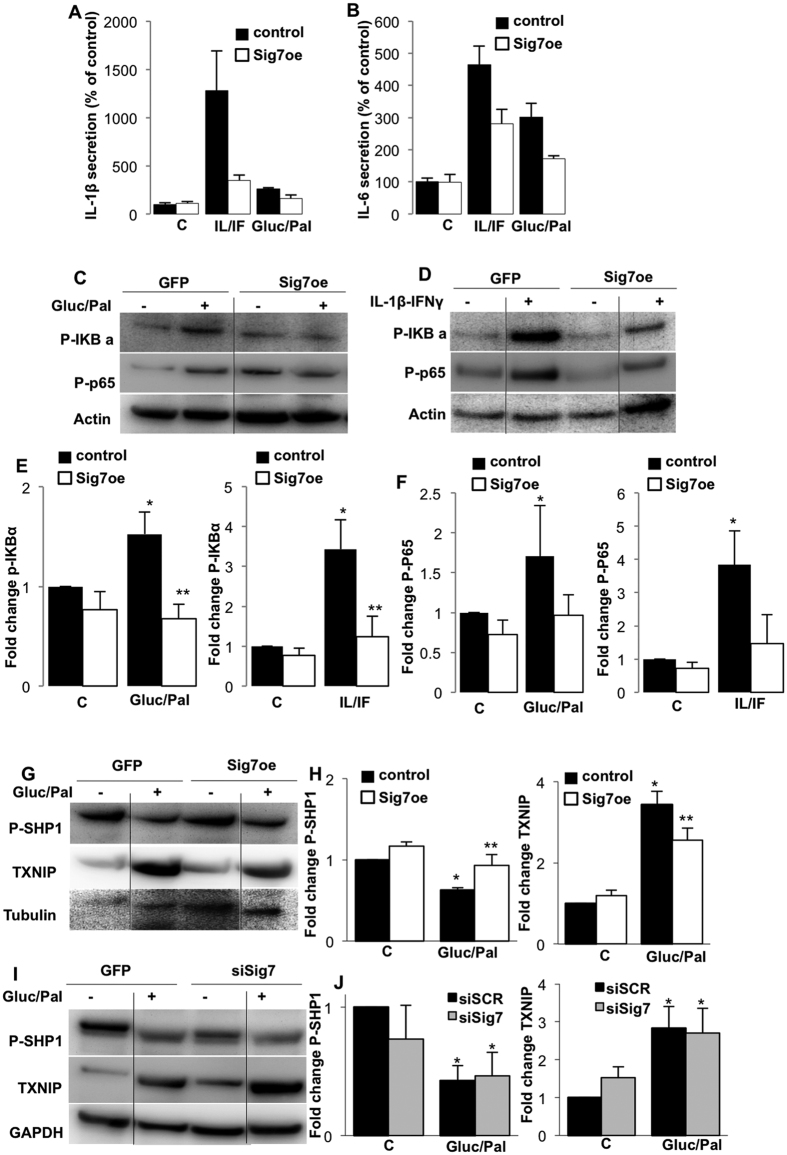
Siglec-7 inhibits NF-κB activation and cytokine secretion by pancreatic islets. Human pancreatic islets were cultured on extracellular matrix-coated dishes and exposed to elevated glucose (22.2 mM) and palmitate (Gluc/Pal) or the cytokine mixture IL-1β (2 ng/ml) and IFNγ (1,000 U/ml) (IL/IF) for 72 h with or without over-expression of lipofectamine-mediated Siglec-7 plasmid transfection. (**A,B**) The cytokine profiles of the supernatants of transfected and treated islets were assessed using protein array ELISAs for IL-1β and IL-6; absolute values at control are 4.2 + /−4.75 pg/ml IL-1β and 458 + /−51 pg/ml IL-6. (**C–F**) Western blot analysis was performed after Siglec-7 over-expression in islets and 72 h treatment with 22.2 mM glucose and palmitate or IL-1β and IFNγ; and analyzed for P-p65, P-IKBα and actin. (**G–J**) Western blot analysis of P-SHP1 and TXNIP from isolated human islets overexpressing Siglec-7 or after Siglec-7 silencing and exposure to 22.2 mM glucose and palmitate for 72 h. (**E,F,H,J**) Densitometry analysis of bands normalized on housekeeping proteins and plotted as fold change of islets at control condition. (**A,B**) are means of 2 independent experiments from 2 different organ donors from 6 dishes per treatment condition. All blots are representative of 3–5 independent experiments and densitometry are means of 3–5 independent experiments from 3–5 different organ donors. Lanes were run on the same gel but were noncontiguous. Full blots are shown in the Suppl. Fig. section “Full blots”. *p < 0.05 to 5.5 mM glucose non-treated control islets, **p < 0.05 Siglec-7 vs. LacZ transfected islets under same diabetic stimuli.

To further define the mechanisms of the protective effects of Siglec-7 overexpression, islets were exposed to elevated glucose and palmitate or to cytokines. As Siglec-7 is an inhibitory molecule on immune cells^37^, we hypothesized that the protective role of Siglec-7 might be mediated via the inhibition of inflammation. Expression and secretion of both IL-1β and IL-6 were induced by exposure of islets to elevated glucose and palmitate as well as by the cytokine mixture IL-1β/IFNγ itself; such induction in cytokine release was reduced in islets over-expressing Siglec-7 (Fig. 4A,B), also the self-induction of its own release was inhibited by Siglec-7 overexpression, suggestive of a direct effect of siglec mediated signalling on cytokine production.

In order to identify the underlying signaling cascades of Siglec-7 mediated β-cell protection, downstream inflammatory pathways were investigated. Western blot analysis of isolated islets treated with elevated glucose and palmitate or the cytokine mixture revealed the activation of the NFκB pathway, as observed by the induction of phosphorylation of IκB-α and p65 phosphorylation at Ser536, both core components leading to NFκB activation. Such induction did not occur in islets which over-express Siglec-7 (Fig. 4C–F). Also, chronic exposure of islets to a diabetic milieu resulted in diminished phosphorylated SHP1, which was restored upon Siglec-7 overexpression (Fig. 4G,H), providing evidence of engagement and activation of Siglec-7. Siglec-7 induced induction of SHP1 phosphorylation was also confirmed in HEK293 cells (data not shown).

Under these conditions, the regulation of the thioredoxin-interacting protein (TXNIP) was also analyzed. Under diabetogenic conditions and oxidative stress, TXNIP binds NALP3 leading to its activation and subsequent IL-1β maturation and secretion^38^. TXNIP was induced upon exposure to diabetogenic stimuli, which could be prevented by Siglec-7 overexpression (Fig. 4G,H). Depletion of Siglec-7 itself had only a moderate effect on SHP and TXNIP and did not further potentiate TXNIP increase or SHP loss under gluco/lipotoxicity (Fig. 4I,J).

These observations consolidated the immunosuppressive role of Siglec-7 in the prevention of triggering islet inflammation observed in diabetes.

### Loss of Siglec-7 is a feature of activated monocytes and overexpression of Siglec-7 inhibits immune cell migration into inflamed islets

**Figure 5:**
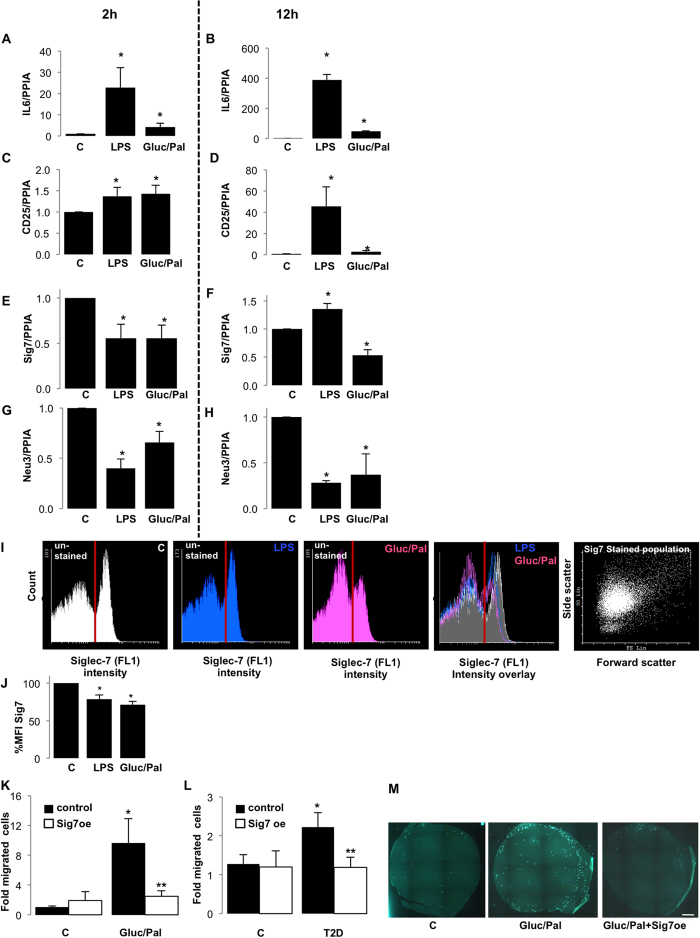
Immune cell migration into inflamed islets is inhibited by Siglec-7. PBMCs purified from buffy coats of blood donors (n = 6) were treated with lipopolysaccharide (LPS) or elevated glucose and palmitate (Gluc/Pal) for (**A,C,E,G**) 2 h or (**B,D,F,H**) 12 h. Real time PCR analysis of these treated cells was carried out for (**A,B**) IL-6, (**C,D**) CD25, (**E,F**) Siglec-7 and (**G,H**) Neu-3. (**I**) Cell surface expression of Siglec-7 in the treated PBMCs was determined using flow cytometry. Histograms for intensity of Siglec-7 (FL1 filter) was plotted and overlayed to observe the effect of these treatments. (**J**) Histograms were quantified and Siglec-7 expression was plotted as % mean fluorescent intensity as compared to untreated control fraction. The migration of leukocytes (n = 3 buffy coat donors) in response to conditioned media obtained from transfected and treated islets (n = 3 separate dishes from 3 independent experiments from 3 donors), was quantified after 4 h using an *in vitro* migration assay. (**K**) The fold induction of migration as compared to untreated control islet supernatants was plotted. (**L**) Migration of mononuclear cells (n = 3) with respect to cultured islets from donors with T2D (n = 3), with or without Siglec-7 over-expression, was plotted as fold change of migrated cells compared to untreated control islets of a non-diabetic individual. (**M**) The images are representative of fluorescent microscopic analysis of live cells migrating through membranes observed in green (shown in (K)). For PBMC treatments; *p < 0.05 to 11.1 mM glucose treated monocyte fraction. For migration assay, *p < 0.05 to monocyte fraction treated with 5.5 mM glucose, LacZ transfected control islets, **p < 0.05 to monocyte fraction treated with Gluc/Pal, LacZ transfected control islets.

Siglec-7 is classically expressed by cells of the innate immune system, and PBMCs also showed NFκB activation by a diabetogenic milieu. Thus, we subsequently investigated whether diabetogenic conditions would also affect Siglec-7 expression in enriched monocytes. The monocyte-enriched fraction of peripheral blood mononuclear cells (PBMCs) was isolated from human buffy coats using sequential Ficoll-Percoll gradients and exposed to control media, lipopolysaccharide (LPS, 20 μg/ml) as known activator of the immune cells or the mixture of 22.2 mM glucose and 0.5 mM palmitate. The activated state of these cells was analyzed after 2 h and 12 h by mRNA analysis of CD25 and IL-6. LPS elicited rapid (after 2 h) and sustained (after 12 h) activation of immune cells, as seen by the induction of IL-6 expression (Fig. 5A, B; ~22-fold/~390-fold at 2 and 12 h as compared to control). Elevated glucose/palmitate also induced monocyte activation, but to a lesser extent (Fig. 5A,B; ~4-fold/~50-fold at 2 and 12 h as compared to control). Both LPS and glucose/palmitate induced the expression of CD25 after 2 h and, similar to IL-6, CD25 expression was induced to a much greater extent after 12 h, with LPS again showing a stronger effect (~1.37-fold, ~1.43-fold at LPS and Gluc/Pal after 2 h; Fig. 5C and ~45-fold, ~3-fold at LPS and Gluc/Pal at 12 h respectively; Fig. 5D, as compared to control). In parallel, Siglec-7 mRNA expression was down-regulated within 2 h of treatment with LPS or glucose/palmitate (Fig. 5E, 45% reduction as compared to control). While LPS-induced Siglec-7 down-regulation was only transient, glucose/palmitate induced a sustained Siglec-7 down-regulation (Fig. 5F; 47% reduction as compared to control), suggesting that chronic activation occurs under conditions of glucolipotoxicity. In line with our findings that Neu3 is decreased in islets in T2D, there was marked down-regulation of Neu3 gene expression in the activated PBMCs at both acute and chronic treatments (Fig. 5G,H). Flow cytometric analysis of treated PBMCs for 2 h confirmed the decrease in Siglec-7 (Fig. 5I). The mean fluorescence intensity of Siglec-7 staining was decreased by 21.38% by LPS and by 28.49% by glucose/palmitate, as compared to control (Fig. 5J), along with increased expression of CD25 (Suppl. Fig. 3A).

To assess the activated cell population, cell surface expression of CD25 and CD14 was analysed along with Siglec-7. By plotting the intensity of Siglec-7 vs. intensity of either CD14 or CD25, followed by quadrant analysis, we determined the Siglec-7 intensity in these cells. The size of the CD25 and Siglec-7 population increased upon exposure to LPS or Gluc/Pal (Suppl. Fig. 3C), but Siglec-7 expression on these cells decreased (Suppl. Fig. 3D). On the other hand the number of CD14^+^Sig7^+^ cells showed a decline under Gluc/Palm treatment (Suppl. Fig. 3E), with Siglec-7 expression remaining stable in these cells (Suppl. Fig. 3F). Thus, loss of Siglec-7 expression was observed only in activated PBMCs, indicating the decreased immune-suppression in these conditions.

Increased infiltration of macrophages has been observed in islets in T2D^39^. To elaborate on the immune-regulatory role of Siglec-7, we evaluated the migration of monocytes *in vitro* in response to conditioned media obtained from isolated human islets, which had been exposed to diabetogenic conditions. For this, we established an *in vitro* migration assay wherein leukocytes isolated from human buffy coats were allowed to migrate over a period of 4 h through a membrane to the lower compartment containing the conditioned islet media. The membranes were mounted in fluorescein diacetate solution, which rendered the live cells fluorescent upon excitation and enabled quantification by fluorescence microscopy (Fig. 5K–M). Induced migration of immune cells was observed under conditions of elevated glucose/palmitate (Fig. 5K, 9.61-fold as compared to control), which demonstrates the triggering of inflammation in islets upon chronic exposure to elevated glucose/palmitate. Conditioned media from islets overexpressing Siglec-7 markedly inhibited the migration of immune cells (Fig. 5K, 74% reduction as compared to glucose/palmitate-treated, LacZ-transfected control islets). We also analysed the migratory response of the cells towards isolated islets obtained from patients with T2D. Supernatants from cultured T2D islets induced significantly higher migration of the immune cells (Fig. 5L, ~2.2-fold induction as compared to non-diabetic islet supernatants, p < 0.05), which could also be blocked by restoring Siglec-7 expression in these islets (Fig. 5L; 54%, reduction as compared to LacZ-transfected control T2D islets, p < 0.05). Treatment with glucose and palmitate did not further induce the migration in islets from patients with T2D (data not shown). These findings highlight the anti-inflammatory role and the inhibition of immune cell stimulation by Siglec-7 in β-cells.

### Siglec functional paralogs are absent in mouse endocrine cells

The CD33-related siglecs are a family of evolutionarily non-conserved genes with unique expression patterns observed in humans^15^,^16^,^17^,^18^,^19^,^40^,^41^,^42^,^43^, and even chimpanzees do not express all the same Siglecs^18^,^43^. In confirmation with such previous data, we found the siglec functional paralogs absent in mouse endocrine cells. FACS staining of isolated and dispersed islet cells displayed only a very low percentage of Siglec-F positive cells (Suppl. Fig. 4A), which was specific, since the expected signal reduction was observed in islets from Siglec-F knockout mice. Similarly, only a very small population of Siglec-E expressing cells was found (Suppl. Fig. 4B). Antibody specificity of Siglec-E and –F antibodies was confirmed by flow cytometric analysis of HEK293 cells overexpressing Siglec-E and -F (Suppl. Fig. 5). Because of the small amount of Siglec-E and –F positive cells we wondered, which islet cells express the siglecs and whether the low signal comes from resident macrophages within the islets. To deplete the macrophages, isolated wildtype islets were treated with clodronate containing liposomes^44^, which reduced CD68, F4/80 as well as CD11b expression significantly (Suppl. Fig. 4C). Along with a reduction in the macrophage markers, Siglec-E was also strongly and significantly reduced and Siglec-F was not detected after macrophage depletion (Suppl. Fig. 4E), while β-cell markers remained unchanged (Suppl. Fig. 4D). As expected, cytokine expression was also affected by clodronate treatment. While IL-6 did not change, IL-1β was significantly reduced compared to PBS control, suggesting resident macrophages as the main source of IL-1β release (Suppl. Fig. 4E). Also Siglec-G and –H mRNA showed very low expression levels in islets (not shown). Consequently, Siglec-F^−/−^ mice presented no metabolic phenotype, neither in control nor in streptozotocin (STZ) induced diabetic mice. 8-week old male Siglec-F^−/−^ mice and their heterozygous and wildtype littermates were injected with 50 mg/kg STZ on 5 consecutive days to induce diabetes (multiple low dose STZ model). STZ injected mice showed higher random blood glucose levels (Suppl. Fig. 4F), reduced glucose tolerance (Suppl. Fig. 4G), impaired insulin secretion (Suppl. Fig. 4H,I) as well as lower β-cell mass (Suppl. Fig. 4J), but no differences were detectable between the different genotypes (Suppl. Fig. 4F–J,). Also in isolated wildtype, Sig-F^+/−^ and Sig-F^−/−^ islets, Siglec-F depletion had no effect on insulin secretion, neither at 5.5 mM glucose control nor under diabetogenic conditions (72 h high glucose/palmitate or cytokine mixture; Suppl. Fig. 4K).

## Discussion

In the present study we show the expression of several CD33-related subset of siglecs in specific endocrine cells of pancreatic islets. Siglecs are a novel family of signalling molecules, originally thought to be primarily expressed in hematopoietic cells. While other exceptions have been reported^18^ we now demonstrate their presence in islets for the first time and show that their levels are regulated in diabetes. Of the evolutionarily evolving CD33-related siglecs, which were investigated, Siglec-7 and -10 were expressed solely in islet β-cells, whereas Siglec-3, -5 and -8 were expressed only in α-cells. Although Siglec-7 shares around 84% sequence homology with Siglec-9^45^, the latter was undetectable in the endocrine pancreas as assessed by real-time PCR and immunohistochemical analyses. The β-cell specific Siglec-7 was markedly down-regulated in pancreas of individuals with T2D. It also appeared that Siglec-7 was downregulated in cases of recent-onset T1D, since β-cells were still present but Siglec-7 expression was barely detectable.

In view of the marked regulation of Siglec-7 in diabetes, we focused our attention on its role in the β-cells. A decrease in the surface expression of Siglec-7 was described as a principal marker of the aberrant NK-cell dysregulation in patients with chronic HIV-1 viremia^46^. Thus, we hypothesized that the loss of Siglec-7 in β-cells might contribute to their dysfunction and apoptosis in diabetes. Indeed, restoring surface Siglec-7 expression protected the β-cells from the deleterious effects of a diabetic milieu. Siglec-7 not only maintained glucose stimulated insulin secretion under diabetogenic conditions, but it also inhibited β-cell apoptosis. This rescue of function and survival was also evident in *in vitro* studies of freshly isolated islets from patients with T2D. Additionally, depletion of Siglec-7 in isolated islets from controls also impaired β-cell function. This was mainly due to an increase in basal insulin and suggests insulin release from apoptotic cells at low glucose concentrations, which leads to a loss in glucose response. This was further potentiated under cytokine conditions. Although apparent in all experiments, the effect of Siglec downregulation by siRNA had a rather modest effect of β-cell function and survival. Similarly, at basal conditions, Siglec-7 overexpression shows no effects, and only becomes apparent at diabetogenic conditions, where survival and function could be restored.

Siglec-3 also has cytoplasmic ITIMs, but its functional significance in α-cells needs to be further investigated. Also, since it is one C2-set domain shorter than Siglec-7, this might lead to alterations in its interaction partners within the islets.

Siglec-7 has an unusual binding preference for α2,8-linked disialic acids and weaker interactions with branched α2,6-sialyl residues^32^. Thus, we investigated the presence of these ligands in the human pancreas by immunohistochemistry using Siglec-7 Fc-chimeras, and found them to be expressed in islets. Interaction partners were present in both α- and β-cells, indicating the possibility of intra-islet *trans* interactions of Siglec-7 with its ligands on both of these cell types. In line with this, an endogenous ligand of Siglec-7, the ganglioside GD3, was strongly up-regulated in islets in diabetes. The disialoganglioside GD3 is an acidic glycosphingolipid, generated downstream of the ceramide-driven ganglioside biosynthesis, by sialylation of its immediate precursor GM3 by GD3 synthase (α2,8-sialyltransferase or St8Sia1 or SAT II)^47^. In freshly isolated islets of patients with T2D, we detected increased St8Sia1 expression, which supports our observation of up-regulated GD3 in diabetes.

Such increased St8Sia1 confirms a very recent study showing higher St8Sia1 protein in T2D islets^33^. Also in this study, a high variation was observed among the T2D islets. St8Sia1 positive β-cells are less functional^33^ and probably contribute to the dysregulation of β-cell function in diabetes. While St8Sia1 is responsible for the Siglec-7 specific α2,8 linkage formation, the Siglec-7 ligand GD3 is the ganglioside that it synthesizes.

GD3 activates Fas and ceramide mediated apoptosis, directly targets mitochondria and disrupts mitochondrial trans-membrane potential^48^, leading to the release of pro-apoptotic factors such as cytochrome c, production of ROS and activation of AIF and caspase-9^49^. Its induced expression in diabetes, thus, hints not only towards feedback up-regulation of ligand upon loss of Siglec-7 expression, but also provides indirect evidence for activation of pro-apoptotic signaling via the Fas receptor, previously reported in the context of glucotoxicity as well as immune mediated β-cell destruction in islets^50^,^51^. Increased levels of GD3 in serum have also been implicated in inflammatory processes such as atherosclerosis^52^ and lipopolysaccharide triggered inflammation in brain, wherein the microglial cells are activated and secrete GD3 leading to apoptosis of oligodendrocytes^53^. Parallels can be drawn between cytokines (e.g. IL-1β) and GD3, as at low concentrations, both stimulate cell proliferation while at higher concentrations they trigger apoptosis^9^,^54^. Also, GD3 expression has been observed specifically in the islets of the NOD mouse model of T1D, whereas its precursor GM3 is expressed in wild type islets^55^. Hence, increased GD3 expression reinforces the pro-apoptotic inflamed state of islets in T2D. Further studies of 9-O-acetyl GD3^56^ are currently being pursued by others, as this modified form can have opposing functions^57^.

While we observed increased GD3 synthase, the membrane-associated sialidase specific for ganglioside, Neu3^58^ was decreased in islets isolated from individuals with T2D. Neu3 cleaves the surface sialic acid residues on ganglioside, which can unmask Siglec-7 and induce its inhibitory signaling cascade^25^. This unmasking may be diminished in T2D, as observed by significantly lower levels of Neu3 leading to increased Siglec-7 ligand expression in islets from patients with T2D. Tissue specific effects of Neu3 were observed previously; mice over-expressing Neu3 mainly in muscles develop severe insulin-resistant diabetes^59^, but, hepatic Neu3 over-expression improves insulin sensitivity and glucose tolerance through modification of ganglioside composition and Peroxisome Proliferator-activated Receptor gamma (PPAR-γ) signaling^60^. In islets, PPAR-γ activation restores β-cell function under conditions of hyperglycemia and cytokine stress^61^ and also regulates the β-cell transcription factors PDX-1 and Nkx6.1^62^. Decreased NEU3 expression in T2D islets may thus lead to reduced PPAR-γ signaling, and hence might contribute to β-cell dysfunction under diabetogenic conditions.

Activated immune cells are present in obesity and promote insulin resistance^63^. Chronic exposure to FFAs leads to activation of monocytes, along with up-regulation of toll-like receptors TLR2 and TLR4^64^, which leads to impaired glucose metabolism on the level of insulin sensitive as well as insulin producing cells. As Siglec-7 is endogenously expressed mainly by natural killer cells and monocytes and balances the immune response, the observed loss of Siglec-7 in blood monocytes was a hallmark of activated monocytes under diabetogenic conditions. Acute exposure to LPS as well as elevated glucose and palmitate was sufficient to inhibit Siglec-7 expression. In spite of this, chronic exposure to LPS led to restoration of the messenger RNA, but the cell surface expression of Siglec-7 was still reduced. Glucose and palmitate chronically maintained both the mRNA and protein at a low level, consistent with a chronic effect on the activation of the immune cells under circumstances when IL-6 was also induced. CD25, an atypical marker for activated macrophages^65^ was induced after chronic exposure to the diabetic milieu, supporting the activation of these cells.

The down-regulation of Siglec-7 in activated PBMCs goes hand in hand with the decreased expression found in islets under conditions of inflammation, which highlights its potential anti-inflammatory role in both of these cell types. Ultimately, we investigated the effect of restoration of siglecs in the islets on the infiltration of the immune cells.

Maintaining Siglec-7 expression in stressed islets under glucolipotoxic conditions inhibited the recruitment and migration of the immune cells. The increased number of macrophages per islet observed *in vivo* in diabetes^39^ was confirmed *in vitro* using a leukocyte migration assay. Moreover, increased leukocyte infiltration was also observed into islets from patients with T2D as well as under diabetogenic conditions *in vitro*, both which were prevented by restoring Siglec-7 expression in these islets. Hence, Siglec-7 expression in islets is essential for maintaining an anti-inflammatory environment in islets, which prevents apoptotic signals and subsequent immune system activation. The major apoptotic pathway JNK, which activation determines the switch towards severe apoptosis in human islets^66^,^67^, was also influenced by Siglec-7 overexpression. While cytokine treatment induced induction of PARP and Caspase 3 cleavage and JNK phosphorylation in the human β-cell line CM9^68^, Siglec-7 overexpression could reduce apoptosis as well as p-JNK (data not shown).

In this paper, the role of Siglec-7 in diabetes has been further investigated *in-vivo* due to the high inter-species variation in the structure and function of CD33-related siglecs. It was suggested that human CD33-related siglecs have arisen due to repeated gene duplication, exon shuffling and gene conversions on chromosome 19^20^,^69^ leading to >10 siglec genes, and many pseudogenes. In syntenic region in mice, CD33-related siglec gene clusters are localized on chromosome 7 with just 4 siglec genes and fewer pseudogenes^70^. Functional and structural orthologous of siglecs are poorly understood and one-to-one orthologue determination is not feasible as seen by phylogenetic analysis of human and mouse gene clusters and whole genome analysis comprising of study of gene structure and localization on chromosome^70^. Hence, it is difficult to extrapolate from mouse to human CD33-related Siglec studies. In contrast to the endocrine expression of human siglecs in the exocrine pancreas, among the evolutionarily evolving CD33-related siglecs, murine orthologues Siglec-E and Siglec-F expression in islets was restricted to resident macrophages. Resident macrophages are maintained in islet cell culture, seen by macrophage markers in mouse and human islets^44^,^71^ and also confirmed in this study.

A mouse knockout of Siglec-F was nevertheless subjected to functional *in vivo* studies, but showed no visible effect on insulin secretion and diabetes progression. This difference in siglec expression and function in human and mouse islets makes the mouse an inadequate model for investigating the effect of CD-33 related siglecs in diabetes and thus, true *in vivo* studies are impossible. Further studies are needed in other species to see if the observed islet expression is unique to humans, as unique human siglec expression patterns have been observed before^15^,^16^,^17^,^18^,^19^,^40^,^41^,^42^,^43^.

There are large differences in the cellular Siglec-7 glycosylation patterns^72^. Siglec-7 was found as a 75-kDa protein on NK-cells with a 46-kDa backbone^23^; here we detect a 100 kDa band in Siglec-7 overexpressing CHO cells, while the commonly known Siglec-7 46-kDa band was identified in PBMCs and human islets.

**Figure 6:**
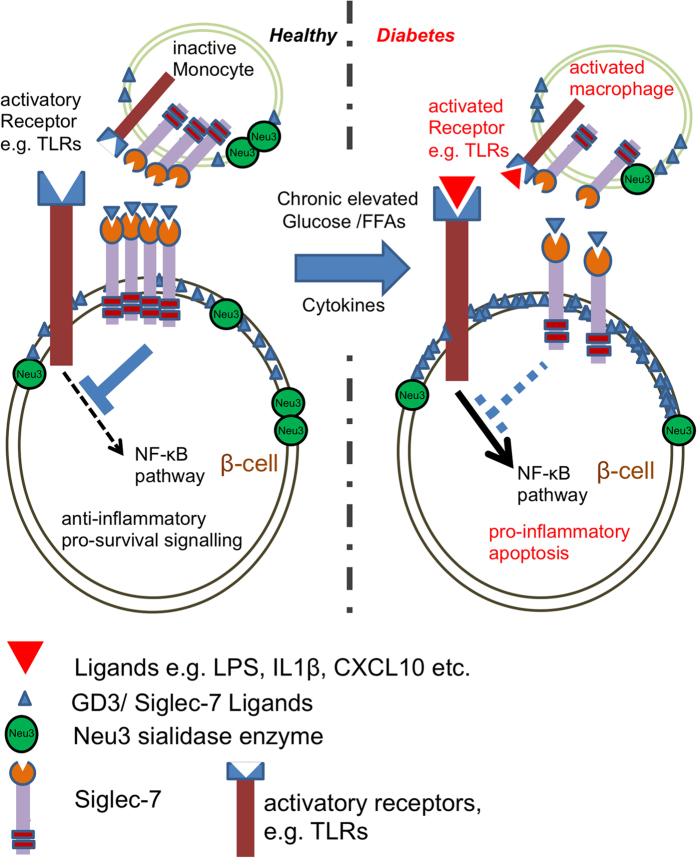
Our view on the deleterious loss of Siglec-7 signals under diabetogenic conditions. In healthy individuals, Siglec-7 helps to maintain a pro-survival anti-inflammatory signaling in monocytes as well as in β-cells. The membrane-associated sialic acid-cleaving enzyme sialidase Neu3 unmasks Siglec-7 residues and makes them free for binding and activation and thus enhances Siglec-7 inhibitory downstream cell protection events (e.g. NFκB inhibition). In diabetes, chronically elevated glucose along with palmitate and cytokines cause loss of Siglec-7 in these cells. The simultaneous loss in the sialidase Neu3 and the increase in endogenous Siglec-7 ligands block siglec downstream signals. This leads to triggering of apoptotic and pro-inflammatory signals, activation of macrophages and ultimately to β-cell death.

Summarizing our findings, we detected the presence of a novel family of cell adhesion molecules- siglecs, expressed in the endocrine cells of the pancreas. One of its β-cell specific members, Siglec-7 was lost in diabetes (Fig. 6). Restoration of Siglec-7 in these cells protected them from the harmful effects of diabetic milieu, and helped to preserve β-cell function and survival under these conditions by inhibition of pro-inflammatory cytokine secretion and suppression of NF-κB activity. Not only was this immune-modulatory function evident in the cytokine profile of human islets, but PBMCs also showed loss of Siglec-7 expression upon activation. Our data suggest that Siglec-7 plays a critical role in the maintenance of an immune-suppressive anti-inflammatory microenvironment, which is lost in diabetes, and may contribute to the manifestation and progression of this metabolic syndrome (Fig. 6). Thus, preserving Siglec-7 expression and function on β-cells as well as on immune cells may be a novel therapeutic strategy which could help target the sensitization and pro-inflammatory activation of the immune system as well as the islets, thereby halting the deterioration of islets in diabetes.

## Experimental Procedures

### Islet culture

Human islets were isolated from ten pancreata of non-diabetic control and 3 organ donors with T2D at the Universities of Lille or Leiden and cultured in CMRL-1066 medium (Invitrogen) as described previously^73^. Briefly, islets were cultured on extracellular matrix coated dishes derived from bovine corneal endothelial cells (Novamed Ltd., Jerusalem, Israel) for 2 days, allowing the cells to attach to the dishes and spread^74^. They were exposed to 5.5, 22.2 or 33.3 mM glucose, with or without 0.5 mM palmitate (dissolved as described previously^75^) or the mixture of 2 ng/ml recombinant human IL-1β (R&D Systems, Minneapolis, MN) + 1,000 U/ml recombinant human IFNγ (PeProTech, Rocky Hill, NJ, USA) for 72 h. For depletion of macrophages mouse islets were cultured in suspension with 0.5 mg/ml clodronate or PBS containing liposomes (supplied by N. van Rooijen, Vrije Universiteit Amsterdam, the Netherlands) for 48 h, washed and plated for further experiments afterwards. CHO cells stably expressing Siglec-7, kindly provided by Paul Crocker, Dundee University, were generated by co-transfection of CHO cells with a 10:1 ratio of HDPUW68 and pcDNA3 as described before^24^. The use of human islets in the experiments have been approved by the “University of Bremen ethical committee” and all experiments were performed in accordance with these guidelines and regulations. Informed consent was obtained from all subjects or their relatives and ethical approval given to the respective institutions. Research with human islets from brain dead donors applies to NIH regulations PHS 398, exemption 4.

### Animal studies

Siglec-F-knockout (Siglec-F^−/−^) mice on a C57Bl/6 genetic background were generated as described before^76^. For MLD-STZ experiments, 8-week-old Siglec-F^−/−^ mice and their heterozygous Siglec-F^+/−^ and WT littermates were injected i.p. with streptozotocin (STZ; 50 mg/kg; Sigma) freshly dissolved in 50 mM sodium citrate buffer (pH 4.5) for 5 consecutive days (referred to as multiple low dose/MLD-STZ). Random blood was obtained from the tail vein of non-fasted mice and glucose was measured using a Glucometer (Freestyle; TheraSense Inc., Alameda, CA). All animals were housed in a temperature-controlled room with a 12-hour light/dark cycle and were allowed free access to food and water in agreement to NIH animal care guidelines of the §8 German animal protection law and all protocols approved by the Bremen Senate. All experiments were performed in accordance with these guidelines and regulations.

For intraperitoneal glucose tolerance tests (ipGTT), mice were fasted 12 h overnight and injected i.p. with glucose (40%; B.Braun, Melsungen, Germany) at a dose of 2 g/kg body weight. Blood samples were obtained at time points 0, 15, 30, 60, 90, and 120 min for glucose measurements using a Glucometer. For glucose stimulated insulin secretion mice were fasted for 12 h overnight and blood samples were collected retrobulbar at timepoints 0 and 15 min for measurement of serum insulin levels. Insulin secretion was measured before (0 min) and after (15 min) i.p. injection of glucose (2 g/kg) and measured using ultrasensitive mouse ELISA kit (ALPCO Diagnostics, Salem, NH).

### Transfection

At 2 days post-isolation and culture on extracellular matrix coated dishes, isolated islets were transfected using Ca^2+^-KRH medium (KCl 4.74 mM, KH_2_PO_4_ 1.19 mM, MgCl_2_6H_2_O 1.19 mM, NaCl 119 mM, CaCl_2_ 2.54 mM, NaHCO_3_ 25 mM, HEPES 10 mM). After 1 h incubation lipoplexes (Lipofectamine2000, Invitrogen, Carlsbad, CA, USA)/DNA ratio 2.5:1, 5 μg CMV-Siglec-7 (Life technologies), mouse Siglec-F (BD Bioscience, San Jose, CA, USA #552125), Siglec-E (R&D Systems #AF5806) or LacZ/GFP control plasmid DNA/100 islets or 100 nM siRNA to Siglec-7 (ON-TARGETplus SMARTpool against human *Siglec-7*, (Dharmacon, Lafayette CO, USA) and scramble siRNA (Dharmacon) were added to transfect the cells as described previously^67^. After additional 6 h incubation, CMRL 1066 medium containing 20% FCS and L-Glutamine were added to the transfected islets. Transfection efficiency was determined using RT-PCR.

### Glucose stimulated insulin secretion

Islets used to perform glucose-stimulated insulin secretion experiments were kept in culture medium on matrix-coated plates. For acute insulin release in response to glucose, islets were washed and pre-incubated (30 min) in Kreb’s Ringer bicarbonate buffer (KRB) containing 2.8 mM glucose and 0.5% BSA. KRB was then replaced by KRB 2.8 mM glucose for 1 h (basal), followed by an additional 1 h in KRB 16.7 mM glucose (stimulated). Islet insulin was determined using mouse insulin ELISA (ALPCO, Salem, NH, USA).

### RNA extraction and RT-PCR analysis

Total RNA was isolated from cultured human islets as described previously^73^. For gene expression analysis of siglecs, semi-quantitative Real Time-PCR was performed in the StepOne Plus Real Time PCR system (Applied Biosystems, Darmstadt, Germany) using Power SYBR Green PCR Master Mix (Applied Biosystems, Darmstadt,Germany). cDNA based on RNA from human pancreatic tissues were analyzed for the genes cyclophilin, glucagon, SAT2, insulin and SN1. Amplification of the endogenous housekeeping gene cyclophilin as well as the genes glucagon, SAT2, insulin and SN1 consisted of an initial denaturation step at 95 °C for 10 min, followed by 40 PCR cycles of denaturation by 95 °C for 30 s, primer annealing by 60 °C for 20 s, and elongation by 72 °C for 10 s. All siglecs were amplified carrying out the touchdown PCR with annealing temperatures from 57 °C-53 °C in each 5 cycles. Primers used for this RT-PCR were: (Siglec-7) 5′AAGAAGCCACCAACAATGAG3′/5′CAGTTAGACAAGAGGAATAAGTTC3′; (Siglec-3) 5′TGGTGTGACTACGGAGAG3′/5′ATGAAGAAGATGAGGCAGAG3′ (Siglec-10) 5′CATTATGCCACGCTCAAC3′/5′TCTTCAACCTCTTACTCTACC3′; (insulin) 5′CTACCTAGTGTGCGGGGAAC3′/5′GCTGGTAGAGGGAGCAGATG3′; (glucagon) 5′CATTCACAGGGCACATTCAC3′/5′CAGCTTGGCCTTCCAAATAA3′; (SN1) 5′TACGACGTGCTATCCAGCAG3′/5′CCAGGATTTTAGGGGTGGAT3′; (SAT2) 5′AGTTGCCTTTGGTGATCCAG3′/5′CAGGACACGGAACCTGAAAT3′ and (PPIA) 5′TACGGGTCCTGGCATCTTGT3′/5′CCATTTGTGTTGGGTCCAGC3′. For analysis of PBMCs and isolated islets, we used the Applied Biosystems StepOne Real Time-PCR system (Applied Biosystems, Carlsbad, CA, USA) with a commercial kit (TaqMan(R) PCR Master Mix; Applied Biosystems). TaqMan(R) Primers used: Siglec-7 (Hs00255574_m1); Siglec-3 (Hs00233544_m1); ST8SIA1 (Hs00268157_m1); PPIA (Hs99999904_m1); CD25 (Hs00907779_m1); Neu3 (Hs00198406_m1), Siglec-E (Mm01205915_g1); Siglec-F (Mm00523987_m1); Siglec-G (Mm00556586_m1), Siglec-H (Mm00618527_m1), CD68 (Mm03047343); CD11b (Mm00434455_m1); F4/80 (Mm00802529_m1); IL1β (Mm00434228_m1); IL6 (Mm00446190_m1); Ins1 (Mm04207513_g1); Ins2 (Mm00731595_gH); Pdx1, (Mm00435565_m1); β-Actin (Mm00607939_s1).

To analyze the relative changes in the gene expression the comparative ΔΔC_t_ method was used. According to this method C_t_ values of the genes of interest were normalized the reference genes. The resulting ΔC_t_ values of any sample were adjusted then to a ΔC_t_ value of the control sample, using the formula 2^−ΔΔCt^ in which ΔCt = Ct_target gene_ −Ct_reference gene_ and ΔΔCt = ΔC_t,sample_ − ΔC_t,control_.

### Western Blot analysis

At the end of the incubation periods, islets, PBMCs and CHO cells were washed in ice-cold PBS and lysed in 40 μl lysis buffer RIPA (20 mM Tris acetate, 0.27 M sucrose, 1 mM EDTA, 1 mM EGTA, 50 mM NaF, 1% Triton X-100, 5 mM sodium pyrophosphate and 10 mM β-glycerophosphate) by repeated rounds of freezing and thawing on ice. Prior to use, the lysis buffer was supplemented with Protease- and Phosphatase-inhibitors (Pierce, Rockford, IL, USA). Protein concentration was measured using BCA assay (Pierce, Rockford, IL, USA). Equivalent amounts of protein from each treatment group were run on a NuPAGE 4–12% Bis-Tris gel (Invitrogen) and electrically transferred onto PVDF membranes. Membranes were incubated with rabbit anti- p-IκBα (Cell Signaling Technology Danvers. MA, USA); rabbit anti-P-p65 rabbit (Cell Signaling Technology Danvers. MA, USA); rabbit anti-TXNIP (Abcam, Cambridge, UK); rabbit anti-pSHP1 (Abcam, Cambridge, UK) rabbit anti-β-tubulin (Cell Signaling Technology, Danvers. MA, USA), polyclonal goat Siglec-7 (R&D Systems; AF1138), rabbit anti-GAPDH (Cell Signaling Technology, Danvers. MA, USA), and rabbit anti-β-actin (Cell Signaling Technology, Danvers. MA, USA) antibodies, followed by horseradish-peroxidase-linked anti-rabbit IgG. Membrane was developed using a chemiluminescence assay system (Pierce) and analyzed using DocIT^®^LS image acquisition 6.6a (UVP BioImaging Systems, Upland, CA, USA). Densitometric analysis of the blots was carried out using Vision Works LS Image Acquisition and Analysis software Version 6.8 (UVP BioImaging Systems, Upland, CA, USA). The gray scale values were normalized on the housekeeping genes as loading controls, and the fold change against control condition was plotted.

### Immunocytochemistry

Pancreas from 7 controls, from 5 patients with T2D were obtained from the National Disease Research Interchange (NDRI) and from 4 patients with T1D from the Glasgow Biobank, approval for the studies were granted by the Ethical Commission of Bremen University. The tissues were fixed in 4% para-formaldehyde overnight and embedded in paraffin. Islets cultured in suspension were washed with PBS and were fixed in Bouin’s (Sigma, Hamburg, Germany) solution for 15 min, resuspended in 2% melted agarose in phosphate buffered saline (PBS), followed by short centrifugation and paraffin embedding. Both, islet agarose pellets and human tissue samples were washed overnight in 70% ethanol followed by dewatering in ethanol and xylol and paraffin embedding using Leica TP1020 tissue processor (Leica, Microsystems, Wetzlar, Germany). 4 μm sections were cut using a microtome and mounted on slides. For immunohistochemical analysis of pancreatic and islet sections, they were deparaffinized and rehydrated by washing twice in toluene for 10 min, respectively, in 100%, 95% and 70% ethanol for 3 min, and then in water for 5 min. Slides were then exposed to antigen-retrieval using Antigen Unmasking Solution (Vector Laboratories, Inc. Burlingame, CA, USA) pre-warmed in a microwave at 600 Watt for 3 cycles each 5 min. and 1 min break in between each cycle. The sections were then cooled to room temperature and permeablized in soaking buffer (0.4% Triton X-100 in TBS) for 30 min. To minimize unspecific binding of antibodies, slides were incubated in blocking buffer containing 0.2% Tween 20, 3% IgG-free bovine serum albumin (BSA), and 0.5% Triton X-100 or 1 h RT. For Fc-chimera staining, Human Fc receptor blocking reagent (MACS #130-059-901, Miltenyi Biotec Inc., Auburn, CA, USA) was used for 15–20 min room temperature. As a negative control for staining with Fc chimera, slides were treated with 10 mU/ml *V. cholerae* sialidase (Roche, Mannheim, Germany) for 2 h at 37 °C. Antibodies used were: polyclonal goat Siglec-7 (R&D Systems; AF1138), polyclonal rabbit anti-Siglec-7 (Abcam, Cambridge, UK); rabbit anti-human Sialoadhesin (Abcam); monoclonal mouse anti-Siglec-7 (kindly provided by Prof. Paul Crocker), polyclonal sheep anti-Siglec-3,-5,-8,-7 and -10 (kindly provided by Prof. Paul Crocker); Siglec-7 Fc-chimera expressed in CHO_Lec1_-cells as described previously^77^; rabbit anti-human CD22 (Abcam); GD3 (R24; Abcam); guinea pig anti-insulin and mouse anti-glucagon (Dako, Hamburg, Germany). Secondary antibodies were against the primary antibody species and were either FITC-, Cy3- or AMCA-conjugated antibodies (Jackson). For bright field staining, the secondary antibodies conjugated to enzyme alkaline phosphatase were used, followed by development with BCPI/NBT substrate (Sigma, Steinheim, Germany) for 15 min at room temperature. After the staining procedures, slides were mounted with Vectashield with 4′6-diamidino-2-phenylindole (DAPI) (Vector Labs) or Glycerogelatin. Fluorescence was analyzed using a Nikon MEA53200 microscope (Nikon GmbH, Dusseldorf, Germany) and images were acquired using NIS-Elements software (Nikon). Intensity and saturation of the staining was measured using Adobe Photoshop^©^ Extended analysis software after an adapted model used by Pham *et al*.^78^. Briefly, the insulin positive area in the green channel was manually marked and the selection was saved. By loading this selection onto the red channel image, the area positive for Siglec-7 or GD3 was assigned and measurements were recorded. The mean gray scale values were termed as the saturation and the integrated density values considered as intensities. Control IgG and secondary antibodies alone were included to rule out non-specific staining. The expression of Siglec-7 was confirmed with 4 different polyclonal and monoclonal antibodies.

For detection of β-cell apoptosis and proliferation, insulin and TUNEL (*In Situ* Cell Death Detection Kit -AP; Roche Diagnostics) or Ki67 staining (Mouse anti-Ki67 7B11, Invitrogen, Camarillo, CA, USA) were performed as described previously^79^.

For β-cell mass analysis, ten sections (spanning the width of the pancreas) per mouse were analyzed. Pancreatic tissue area and insulin-positive area were determined by computer-assisted measurements using a Nikon MEA53200 (Nikon GmbH, Dusseldorf, Germany) microscope and images were acquired using NIS-Elements software (Nikon). Mean percent beta-cell fraction per pancreas was calculated as the ratio of insulin-positive and whole pancreatic tissue area. Beta-cell mass was obtained by multiplying the beta cell fraction by the weight of the pancreas.

### Cytokine quantification

The cell culture supernatants stored at −20 °C were evaluated in a cytokine multiplex array system called Meso Scale Discovery^®^ (Gaithersburg, MD, USA) using a kit (Human Pro-inflammatory II 4-plex assay) read at the Sector Imager 6000^®^ as per manufacturer’s instructions.

### PBMC isolation

The isolation of PBMCs from buffy coats was adapted from Repnik U *et al*.^80^. Briefly, buffy coats were obtained from the Central Institute for Transfusion Medicine, Asklepios Klinik Hamburg (Germany) and WBCs were purified using a Ficoll gradient (GE healthcare, Uppsala, Sweden), a subsequent hyperosmotic Percoll gradient (GE healthcare, Uppsala, Sweden) led to separation of monocytes from lymphocytes and a third iso-osmotic Percoll gradient to monocytes from platelets and dead cells. The pellet obtained after this gradient is the monocyte-enriched fraction, which we refer to as PBMCs. According to the forward and side scatter plots, this fraction contains about 55–80% monocytes along with 20–45% of lymphocytes.

### Flow cytometry

The PBMC fraction was cultured in RPMI supplemented with 10% FCS, 2 mM L-Glutamine and 100 U/ml Penicillin-Streptomycin for 12 hours with or without 22.2 mM glucose and 0.5 mM Palmitate. Human and mouse islets from Siglec-F^−/−^ and their corresponding controls were treated with Accutase (PAA, Cat.-No: L11-007) to reach a single cell state. They were seeded on extracellular-matrix coated dishes and allowed to recover for 24 h. For flow cytometry of PBMCs and islet cells, they were fixed in freshly-prepared 1% paraformaldehyde for 10 min at RT. After washing, PBMCs were incubated in polyclonal rabbit anti-Siglec-7 Ab (Abcam) followed by incubation with FITC/Alexa 488 labeled donkey anti-rabbit secondary antibody (Dako, Hamburg, Germany). Rabbit IgG was used as isotype control. For CD25 and CD14 labeling, the PE conjugated anti-CD25 Antibody (Beckman and Coulter A0 7774) and PE-Cy5 conjugated anti-CD14 (Beckman and Coulter A0 7765) were incubated for 30 min at 37 °C. Islet cells were incubated with monoclonal rat-anti Siglec-F (BD Pharmingen) or polyclonal goat anti-Siglec-E (R&D systems) antibodies, followed by FITC-labeled secondary antibodies (Dako, Hamburg, Germany). The fractions analyzed by FACS were: unstained, control with only secondary, single labeled Siglec-E and Siglec-F, or Siglec-7, CD14 and CD25 and a triple stained fraction. Statistical analysis has been performed on the cell populations in all the different quadrants of the dot plots of CD14 vs. Siglec-7 and CD25 vs. Siglec-7 in the triple stained samples, and the data represented in the graphs signify quantifications of the co-stained populations.

### Migration assay

After processing buffy coats through a single Ficoll gradient, the purified leukocyte fraction is plated on cell culture inserts with 0.1 μm pore size membranes, placed in 24 well plates (BD biosciences). The lower chamber contained 1:10 diluted conditioned media obtained from transfected and treated islets and migration was allowed at 37 °C for 4 hours. Post the duration of migration, the membrane was cut off and mounted on slides in fluorescein di-acetate solution, which renders live cells fluorescent. The slides are analyzed with a Nikon MEA53200 (Nikon GmbH, Dusseldorf, Germany) microscope and images were acquired and quantified using NIS-Elements software (Nikon).

### Statistical analysis

Samples were evaluated in a randomized manner by G.D, who was blinded to the treatment conditions. Data are presented as means + /− SE and were analyzed by Student’s *t-* tests.

## Additional Information

**How to cite this article**: Dharmadhikari, G. *et al*. Siglec-7 restores β-cell function and survival and reduces inflammation in pancreatic islets from patients with diabetes. *Sci. Rep.* **7**, 45319; doi: 10.1038/srep45319 (2017).

**Publisher's note:** Springer Nature remains neutral with regard to jurisdictional claims in published maps and institutional affiliations.

## Supplementary Material

Supplementary Dataset

## References

[b1] Donath M. Y., Storling J., Maedler K. & Mandrup-Poulsen T. Inflammatory mediators and islet beta-cell failure: a link between type 1 and type 2 diabetes. J. Mol. Med. 81, 455–470 (2003). 12879149 10.1007/s00109-003-0450-y

[b2] Tisch R. & McDevitt H. Insulin-dependent diabetes mellitus. Cell 85, 291–297 (1996). 8616883 10.1016/s0092-8674(00)81106-x

[b3] Kurrer M. O., Pakala S. V., Hanson H. L. & Katz J. D. Beta cell apoptosis in T cell-mediated autoimmune diabetes. Proc. Natl. Acad. Sci. USA 94, 213–218 (1997). 8990188 10.1073/pnas.94.1.213 PMC19288

[b4] Mokdad A. H. . Prevalence of obesity, diabetes, and obesity-related health risk factors, 2001. JAMA 289, 76–79 (2003). 12503980 10.1001/jama.289.1.76

[b5] Neels J. G. & Olefsky J. M. Inflamed fat: what starts the fire? J Clin Invest 116, 33–35 (2006). 16395402 10.1172/JCI27280 PMC1323268

[b6] Festa A., D’Agostino R. Jr., Tracy R. P. & Haffner S. M. Elevated levels of acute-phase proteins and plasminogen activator inhibitor-1 predict the development of type 2 diabetes: the insulin resistance atherosclerosis study. Diabetes 51, 1131–1137 (2002). 11916936 10.2337/diabetes.51.4.1131

[b7] Donath M. Y., Dalmas E., Sauter N. S. & Boni-Schnetzler M. Inflammation in obesity and diabetes: islet dysfunction and therapeutic opportunity. Cell Metab 17, 860–872 (2013). 23747245 10.1016/j.cmet.2013.05.001

[b8] Hotamisligil G. S., Shargill N. S. & Spiegelman B. M. Adipose expression of tumor necrosis factor-alpha: direct role in obesity-linked insulin resistance. Science 259, 87–91 (1993). 7678183 10.1126/science.7678183

[b9] Maedler K. . Glucose-induced beta-cell production of interleukin-1beta contributes to glucotoxicity in human pancreatic islets. J. Clin. Invest 110, 851–860 (2002). 12235117 10.1172/JCI15318 PMC151125

[b10] Larsen C. M. . Interleukin-1-receptor antagonist in type 2 diabetes mellitus. N Engl J Med 356, 1517–1526 (2007). 17429083 10.1056/NEJMoa065213

[b11] Yuan M. . Reversal of obesity- and diet-induced insulin resistance with salicylates or targeted disruption of Ikkbeta. Science 293, 1673–1677 (2001). 11533494 10.1126/science.1061620

[b12] Crocker P. R. . Siglecs: a family of sialic-acid binding lectins. Glycobiology **8**, v (1998). 10.1093/oxfordjournals.glycob.a018832 9498912

[b13] Jandus C., Simon H. U. & von Gunten S. Targeting siglecs–a novel pharmacological strategy for immuno- and glycotherapy. Biochem Pharmacol 82, 323–332 (2011). 21658374 10.1016/j.bcp.2011.05.018

[b14] Itoyama Y. . Immunocytochemical observations on the distribution of myelin-associated glycoprotein and myelin basic protein in multiple sclerosis lesions. Ann Neurol 7, 167–177 (1980). 6154440 10.1002/ana.410070212

[b15] Ali S. R. . Siglec-5 and Siglec-14 are polymorphic paired receptors that modulate neutrophil and amnion signaling responses to group B Streptococcus. J Exp Med 211, 1231–1242 (2014). 24799499 10.1084/jem.20131853 PMC4042635

[b16] Brinkman-Van der Linden E. C. . Human-specific expression of Siglec-6 in the placenta. Glycobiology 17, 922–931 (2007). 17580316 10.1093/glycob/cwm065

[b17] Wang X. . Expression of Siglec-11 by human and chimpanzee ovarian stromal cells, with uniquely human ligands: implications for human ovarian physiology and pathology. Glycobiology 21, 1038–1048 (2011). 21467073 10.1093/glycob/cwr039 PMC3130538

[b18] Schwarz F., Fong J. J. & Varki A. Human-specific evolutionary changes in the biology of siglecs. Adv Exp Med Biol 842, 1–16 (2015). 10.1007/978-3-319-11280-0_1 25408333

[b19] Mitra N. . SIGLEC12, a human-specific segregating (pseudo)gene, encodes a signaling molecule expressed in prostate carcinomas. J Biol Chem 286, 23003–23011 (2011). 21555517 10.1074/jbc.M111.244152 PMC3123068

[b20] Crocker P. R. & Varki A. Siglecs in the immune system. Immunology 103, 137–145 (2001). 11412300 10.1046/j.0019-2805.2001.01241.x PMC1783234

[b21] Crocker P. R., Paulson J. C. & Varki A. Siglecs and their roles in the immune system. Nature reviews. Immunology 7, 255–266 (2007). 10.1038/nri2056 17380156

[b22] Ferreira R. C. . A type I interferon transcriptional signature precedes autoimmunity in children genetically at risk for type 1 diabetes. Diabetes 63, 2538–2550 (2014). 24561305 10.2337/db13-1777 PMC4066333

[b23] Falco M. . Identification and molecular cloning of p75/AIRM1, a novel member of the sialoadhesin family that functions as an inhibitory receptor in human natural killer cells. J Exp Med 190, 793–802 (1999). 10499918 10.1084/jem.190.6.793 PMC2195632

[b24] Nicoll G. . Identification and characterization of a novel siglec, siglec-7, expressed by human natural killer cells and monocytes. J Biol Chem 274, 34089–34095 (1999). 10567377 10.1074/jbc.274.48.34089

[b25] Nicoll G. . Ganglioside GD3 expression on target cells can modulate NK cell cytotoxicity via siglec-7-dependent and -independent mechanisms. Eur J Immunol 33, 1642–1648 (2003). 12778482 10.1002/eji.200323693

[b26] Vitale C. . Engagement of p75/AIRM1 or CD33 inhibits the proliferation of normal or leukemic myeloid cells. Proc Natl Acad Sci USA 96, 15091–15096 (1999). 10611343 10.1073/pnas.96.26.15091 PMC24778

[b27] Avril T., Floyd H., Lopez F., Vivier E. & Crocker P. R. The membrane-proximal immunoreceptor tyrosine-based inhibitory motif is critical for the inhibitory signaling mediated by Siglecs-7 and -9, CD33-related Siglecs expressed on human monocytes and NK cells. J Immunol 173, 6841–6849 (2004). 15557178 10.4049/jimmunol.173.11.6841

[b28] Ikehara Y., Ikehara S. K. & Paulson J. C. Negative regulation of T cell receptor signaling by Siglec-7 (p70/AIRM) and Siglec-9. J Biol Chem 279, 43117–43125 (2004). 15292262 10.1074/jbc.M403538200

[b29] Macauley M. S., Crocker P. R. & Paulson J. C. Siglec-mediated regulation of immune cell function in disease. Nature reviews. Immunology 14, 653–666 (2014). 10.1038/nri3737 PMC4191907 25234143

[b30] Gammelsaeter R., Jenstad M., Bredahl M. K., Gundersen V. & Chaudhry F. A. Complementary expression of SN1 and SAT2 in the islets of Langerhans suggests concerted action of glutamine transport in the regulation of insulin secretion. Biochem Biophys Res Commun 381, 378–382 (2009). 19233140 10.1016/j.bbrc.2009.02.062

[b31] Blixt O., Collins B. E., van den Nieuwenhof I. M., Crocker P. R. & Paulson J. C. Sialoside specificity of the siglec family assessed using novel multivalent probes: identification of potent inhibitors of myelin-associated glycoprotein. J Biol Chem 278, 31007–31019 (2003). 12773526 10.1074/jbc.M304331200

[b32] Yamaji T., Teranishi T., Alphey M. S., Crocker P. R. & Hashimoto Y. A small region of the natural killer cell receptor, Siglec-7, is responsible for its preferred binding to alpha 2,8-disialyl and branched alpha 2,6-sialyl residues. A comparison with Siglec-9. J Biol Chem 277, 6324–6332 (2002). 11741958 10.1074/jbc.M110146200

[b33] Dorrell C. . Human islets contain four distinct subtypes of beta cells. Nat Commun 7, 11756 (2016). 27399229 10.1038/ncomms11756 PMC4942571

[b34] Richardson S. J., Willcox A., Bone A. J., Foulis A. K. & Morgan N. G. The prevalence of enteroviral capsid protein vp1 immunostaining in pancreatic islets in human type 1 diabetes. Diabetologia 52, 1143–1151 (2009). 19266182 10.1007/s00125-009-1276-0

[b35] Rapoport E., Mikhalyov I., Zhang J., Crocker P. & Bovin N. Ganglioside binding pattern of CD33-related siglecs. Bioorg Med Chem Lett 13, 675–678 (2003). 12639556 10.1016/s0960-894x(02)00998-8

[b36] Bock N. & Kelm S. Binding and inhibition assays for Siglecs. Methods Mol Biol 347, 359–375 (2006). 17072023 10.1385/1-59745-167-3:359

[b37] Angata T. & Varki A. Siglec-7: a sialic acid-binding lectin of the immunoglobulin superfamily. Glycobiology 10, 431–438 (2000). 10764831 10.1093/glycob/10.4.431

[b38] Zhou R., Tardivel A., Thorens B., Choi I. & Tschopp J. Thioredoxin-interacting protein links oxidative stress to inflammasome activation. Nat Immunol 11, 136–140 (2010). 20023662 10.1038/ni.1831

[b39] Ehses J. A. . Increased number of islet-associated macrophages in type 2 diabetes. Diabetes 56, 2356–2370 (2007). 17579207 10.2337/db06-1650

[b40] Wang X. . Specific inactivation of two immunomodulatory SIGLEC genes during human evolution. Proc Natl Acad Sci USA 109, 9935–9940 (2012). 22665810 10.1073/pnas.1119459109 PMC3382539

[b41] Wang X. . Evolution of siglec-11 and siglec-16 genes in hominins. Mol Biol Evol 29, 2073–2086 (2012). 22383531 10.1093/molbev/mss077 PMC3408085

[b42] Soto P. C., Stein L. L., Hurtado-Ziola N., Hedrick S. M. & Varki A. Relative over-reactivity of human versus chimpanzee lymphocytes: implications for the human diseases associated with immune activation. J Immunol 184, 4185–4195 (2010). 20231688 10.4049/jimmunol.0903420 PMC3085894

[b43] Nguyen D. H., Hurtado-Ziola N., Gagneux P. & Varki A. Loss of Siglec expression on T lymphocytes during human evolution. Proc Natl Acad Sci USA 103, 7765–7770 (2006). 16682635 10.1073/pnas.0510484103 PMC1472519

[b44] Nackiewicz D. . TLR2/6 and TLR4-activated macrophages contribute to islet inflammation and impair beta cell insulin gene expression via IL-1 and IL-6. Diabetologia 57, 1645–1654 (2014). 24816367 10.1007/s00125-014-3249-1

[b45] Zhang J. Q., Nicoll G., Jones C. & Crocker P. R. Siglec-9, a novel sialic acid binding member of the immunoglobulin superfamily expressed broadly on human blood leukocytes. J Biol Chem 275, 22121–22126 (2000). 10801862 10.1074/jbc.M002788200

[b46] Brunetta E. . The decreased expression of Siglec-7 represents an early marker of dysfunctional natural killer-cell subsets associated with high levels of HIV-1 viremia. Blood 114, 3822–3830 (2009). 19710502 10.1182/blood-2009-06-226332 PMC2773483

[b47] Malisan F. & Testi R. GD3 in cellular ageing and apoptosis. Experimental gerontology 37, 1273–1282 (2002). 12470841 10.1016/s0531-5565(02)00138-9

[b48] De Maria R. . Requirement for GD3 ganglioside in CD95- and ceramide-induced apoptosis. Science 277, 1652–1655 (1997). 9287216 10.1126/science.277.5332.1652

[b49] Malisan F. & Testi R. GD3 ganglioside and apoptosis. Biochimica et biophysica acta 1585, 179–187 (2002). 12531552 10.1016/s1388-1981(02)00339-6

[b50] Maedler K. . Glucose induces beta-cell apoptosis via upregulation of the Fas-receptor in human islets. Diabetes 50, 1683–1690 (2001). 11473025 10.2337/diabetes.50.8.1683

[b51] Loweth A. C., Williams G. T., James R. F., Scarpello J. H. & Morgan N. G. Human islets of Langerhans express Fas ligand and undergo apoptosis in response to interleukin-1beta and Fas ligation. Diabetes 47, 727–732 (1998). 9588443 10.2337/diabetes.47.5.727

[b52] Prokazova N. V. & Bergelson L. D. Gangliosides and atherosclerosis. Lipids 29, 1–5 (1994). 8139390 10.1007/BF02537083

[b53] Simon B. M., Malisan F., Testi R., Nicotera P. & Leist M. Disialoganglioside GD3 is released by microglia and induces oligodendrocyte apoptosis. Cell Death Differ 9, 758–767 (2002). 12058281 10.1038/sj.cdd.4401027

[b54] Bhunia A. K., Schwarzmann G. & Chatterjee S. GD3 recruits reactive oxygen species to induce cell proliferation and apoptosis in human aortic smooth muscle cells. J Biol Chem 277, 16396–16402 (2002). 11861654 10.1074/jbc.M200877200

[b55] Dotta F. . Pancreatic islet ganglioside expression in nonobese diabetic mice: comparison with C57BL/10 mice and changes after autoimmune beta-cell destruction. Endocrinology 130, 37–42 (1992). 1727711 10.1210/endo.130.1.1727711

[b56] Chen H. Y., Challa A. K. & Varki A. 9-O-acetylation of exogenously added ganglioside GD3. The GD3 molecule induces its own O-acetylation machinery. J Biol Chem 281, 7825–7833 (2006). 16434401 10.1074/jbc.M512379200

[b57] Mukherjee K. . O-acetylation of GD3 prevents its apoptotic effect and promotes survival of lymphoblasts in childhood acute lymphoblastic leukaemia. J Cell Biochem 105, 724–734 (2008). 18655184 10.1002/jcb.21867

[b58] Miyagi T. . Molecular cloning and characterization of a plasma membrane-associated sialidase specific for gangliosides. J Biol Chem 274, 5004–5011 (1999). 9988745 10.1074/jbc.274.8.5004

[b59] Sasaki A. . Overexpression of plasma membrane-associated sialidase attenuates insulin signaling in transgenic mice. J Biol Chem 278, 27896–27902 (2003). 12730204 10.1074/jbc.M212200200

[b60] Yoshizumi S. . Increased hepatic expression of ganglioside-specific sialidase, NEU3, improves insulin sensitivity and glucose tolerance in mice. Metabolism 56, 420–429 (2007). 17292733 10.1016/j.metabol.2006.10.027

[b61] Kono T. . PPAR-gamma activation restores pancreatic islet SERCA2 levels and prevents beta-cell dysfunction under conditions of hyperglycemic and cytokine stress. Mol Endocrinol 26, 257–271 (2012). 22240811 10.1210/me.2011-1181 PMC3275161

[b62] Moibi J. A. . Peroxisome proliferator-activated receptor-gamma regulates expression of PDX-1 and NKX6.1 in INS-1 cells. Diabetes 56, 88–95 (2007). 17192469 10.2337/db06-0948

[b63] Viardot A. . Obesity is associated with activated and insulin resistant immune cells. Diabetes Metab Res Rev 28, 447–454 (2012). 22492715 10.1002/dmrr.2302

[b64] Dasu M. R. & Jialal I. Free fatty acids in the presence of high glucose amplify monocyte inflammation via Toll-like receptors. American journal of physiology. Endocrinology and metabolism 300, E145–154 (2011). 20959532 10.1152/ajpendo.00490.2010 PMC3023203

[b65] Smith P. D. . Intestinal macrophages and response to microbial encroachment. Mucosal Immunol 4, 31–42 (2011). 20962772 10.1038/mi.2010.66 PMC3821935

[b66] Aikin R., Maysinger D. & Rosenberg L. Cross-talk between phosphatidylinositol 3-kinase/AKT and c-jun NH2-terminal kinase mediates survival of isolated human islets. Endocrinology 145, 4522–4531 (2004). 15242986 10.1210/en.2004-0488

[b67] Ardestani A. . MST1 is a key regulator of beta cell apoptosis and dysfunction in diabetes. Nat Med 20, 385–397 (2014). 24633305 10.1038/nm.3482 PMC3981675

[b68] Baroni M. G. . Beta-cell gene expression and functional characterisation of the human insulinoma cell line CM. J Endocrinol 161, 59–68 (1999). 10194529 10.1677/joe.0.1610059

[b69] Crocker P. R. & Varki A. Siglecs, sialic acids and innate immunity. Trends Immunol 22, 337–342 (2001). 11377294 10.1016/s1471-4906(01)01930-5

[b70] Angata T., Hingorani R., Varki N. M. & Varki A. Cloning and characterization of a novel mouse Siglec, mSiglec-F: differential evolution of the mouse and human (CD33) Siglec-3-related gene clusters. J Biol Chem 276, 45128–45136 (2001). 11579105 10.1074/jbc.M108573200

[b71] Westwell-Roper C. Y., Ehses J. A. & Verchere C. B. Resident macrophages mediate islet amyloid polypeptide-induced islet IL-1beta production and beta-cell dysfunction. Diabetes 63, 1698–1711 (2014). 24222351 10.2337/db13-0863

[b72] Mizrahi S., Gibbs B. F., Karra L., Ben-Zimra M. & Levi-Schaffer F. Siglec-7 is an inhibitory receptor on human mast cells and basophils. J Allergy Clin Immunol 134, 230–233 (2014). 24810846 10.1016/j.jaci.2014.03.031

[b73] Schulthess F. T. . CXCL10 impairs beta cell function and viability in diabetes through TLR4 signaling. Cell Metab 9, 125–139 (2009). 19187771 10.1016/j.cmet.2009.01.003

[b74] Kaiser N., Corcos A. P., Sarel I. & Cerasi E. Monolayer culture of adult rat pancreatic islets on extracellular matrix: modulation of B-cell function by chronic exposure to high glucose. Endocrinology 129, 2067–2076 (1991). 1717241 10.1210/endo-129-4-2067

[b75] Maedler K. . Distinct effects of saturated and monounsaturated fatty acids on beta-cell turnover and function. Diabetes 50, 69–76 (2001). 11147797 10.2337/diabetes.50.1.69

[b76] Zhang M. . Defining the in vivo function of Siglec-F, a CD33-related Siglec expressed on mouse eosinophils. Blood 109, 4280–4287 (2007). 17272508 10.1182/blood-2006-08-039255 PMC1885492

[b77] Attrill H. . The structure of siglec-7 in complex with sialosides: leads for rational structure-based inhibitor design. Biochem J. 397, 271–278 (2006). 16623661 10.1042/BJ20060103 PMC1513286

[b78] Pham N. A. . Quantitative image analysis of immunohistochemical stains using a CMYK color model. Diagn Pathol 2, 8 (2007). 17326824 10.1186/1746-1596-2-8 PMC1810239

[b79] Shu L. . Transcription factor 7-like 2 regulates beta-cell survival and function in human pancreatic islets. Diabetes 57, 645–653 (2008). 18071026 10.2337/db07-0847

[b80] Repnik U., Knezevic M. & Jeras M. Simple and cost-effective isolation of monocytes from buffy coats. J Immunol Methods 278, 283–292 (2003). 12957415 10.1016/s0022-1759(03)00231-x

