# Supplementary material for: Siglec-7 restores β-cell function and survival and reduces inflammation in pancreatic islets from patients with diabetes
Source: Sci Rep. 2017 Apr 5;7:45319. doi: 10.1038/srep45319 (PMC5381285; doi:10.1038/srep45319)

Siglec-7 restores β-cell function and survival and reduces inflammation in pancreatic islets from patients with diabetes

Gitanjali Dharmadhikari *et al.*

Supplementary Data

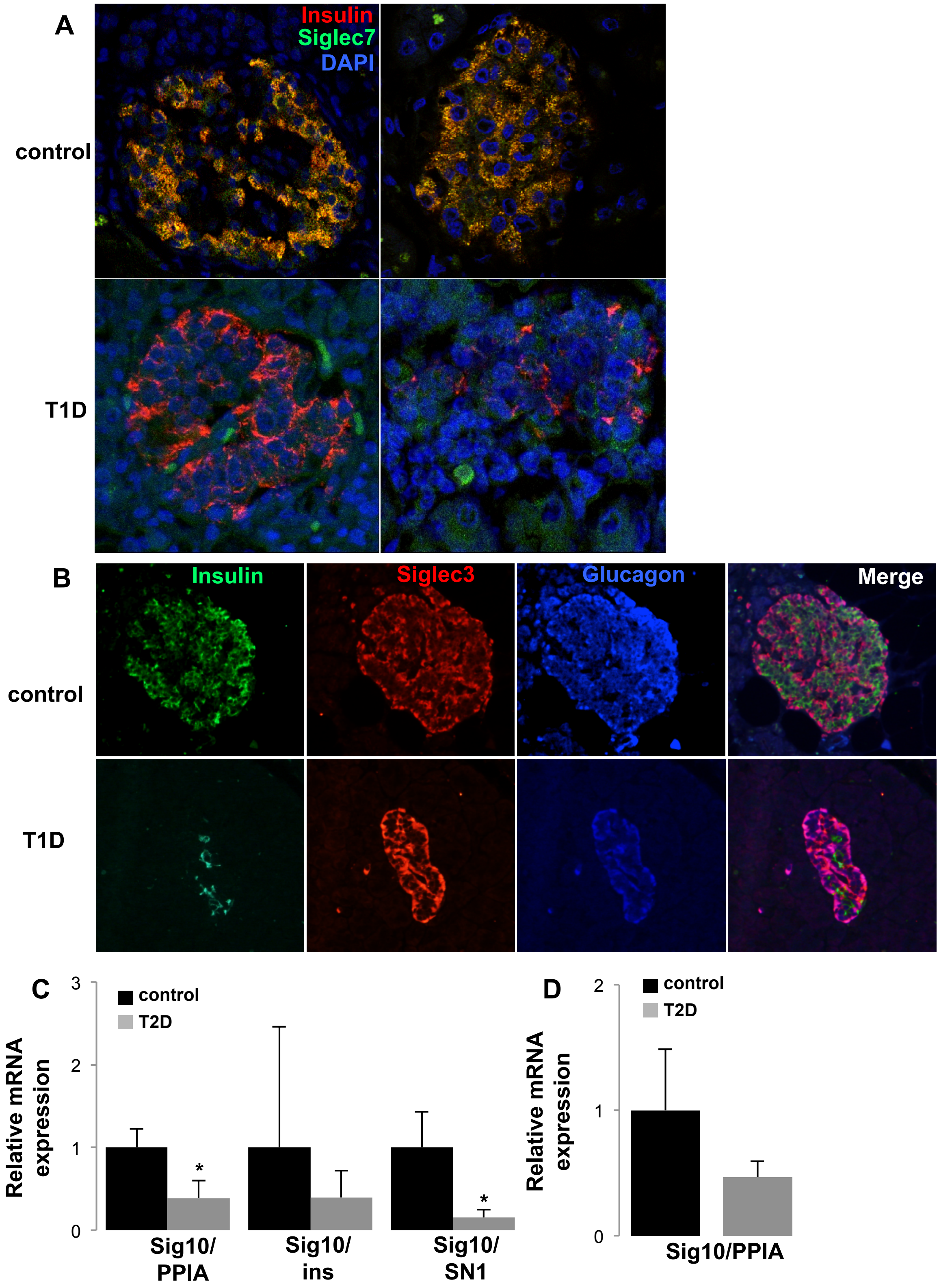

Supplementary Figure 1: Reduced Siglec-7 expression in diabetes.
(A,B) Immunohistochemical analysis was carried out on human pancreatic sections obtained at autopsy of healthy non-diabetic controls and patients with T1D with remaining insulin+ β-cells for Siglec-7 and insulin (confocal microscopy, **A)** and for Siglec-3, insulin and glucagon **(B)**. **(C,D)** Real time PCR analysis was performed on cDNAs obtained from non-diabetic (n=9) and diabetic human pancreas (n=6) from autopsy **(C)** or from freshly isolated islets from T2D patients and nondiabetic control (3 independent experiments from 3 donors, respectively); **(D)** Siglec-10 expression was normalized on cyclophilin (PPIA), insulin and SN1.

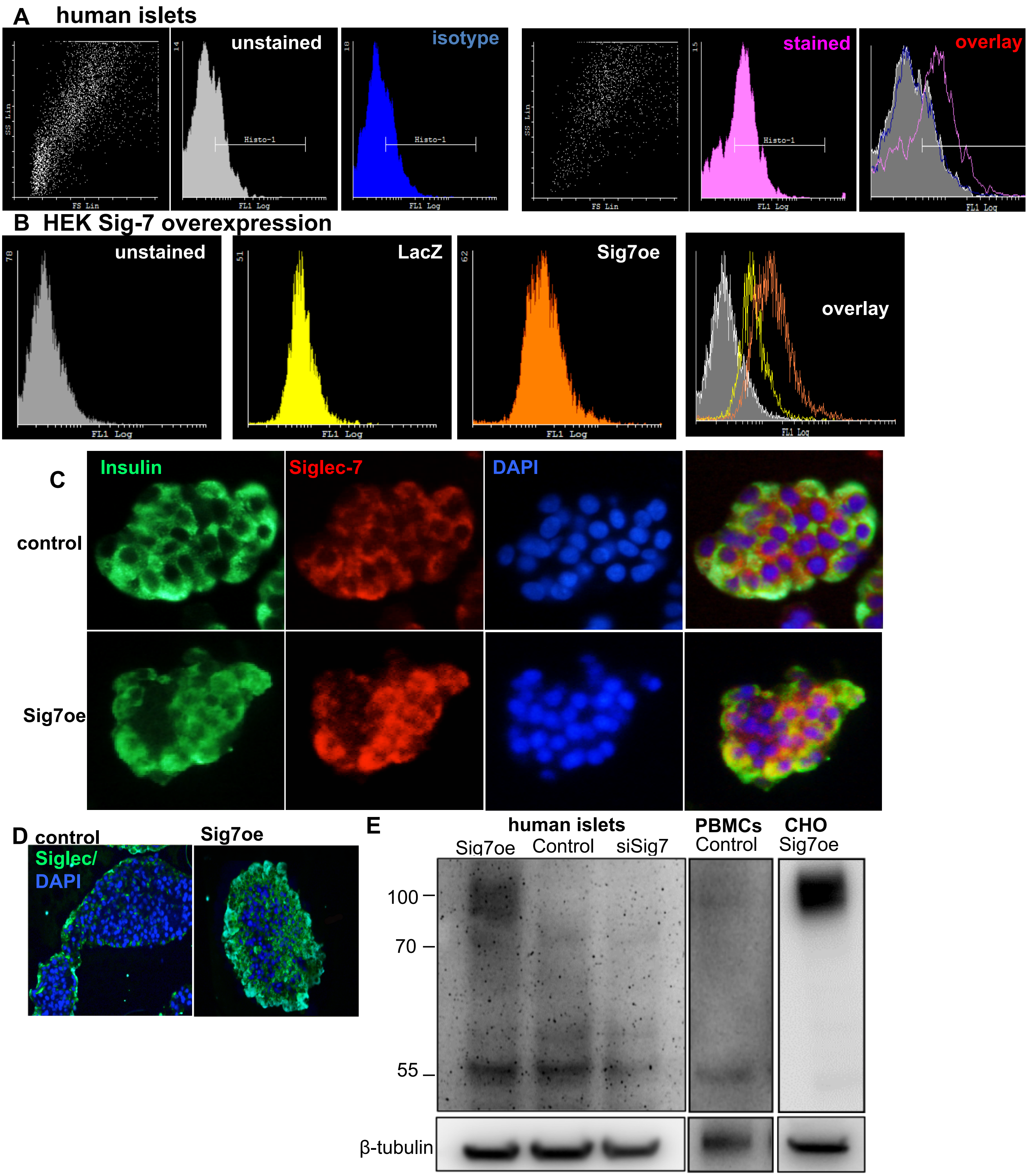

Supplementary Figure 2: **Evaluation of Siglec-7 antibodies. (A)** Human islets were dispersed using accutase and cultured overnight for recovery. These dispersed cells were stained with Siglec-7 and analyzed by flow cytometry. Histograms indicate the intensity of FL1 for unstained, isotype control and Siglec-7 stained cells. **(B)** HEK293T cells were transfected with LacZ or Siglec-7 plasmids, stained for Siglec-7 and analyzed using flow cytometry. **(C,D)** Human Islets were transfected using lipofectamine to overexpress Siglec-7 followed by immunohistochemical analysis of paraffin-embedded islet sections. **(C)** Representative images show insulin (green), Siglec-7 (red) and DAPI (blue) and **(D)** Siglec-7 (green) and DAPI (blue). **(E)** Siglec-7 was analyzed by western blotting using the R&D human Siglec-7 antibody in CHO cells with stable Siglec-7 over-expression, in human PBMCs and dispersed human islets with plasmid Siglec-7 overexpression or siSiglec-7 mediated depletion using 100 nM siRNA to Siglec-7.

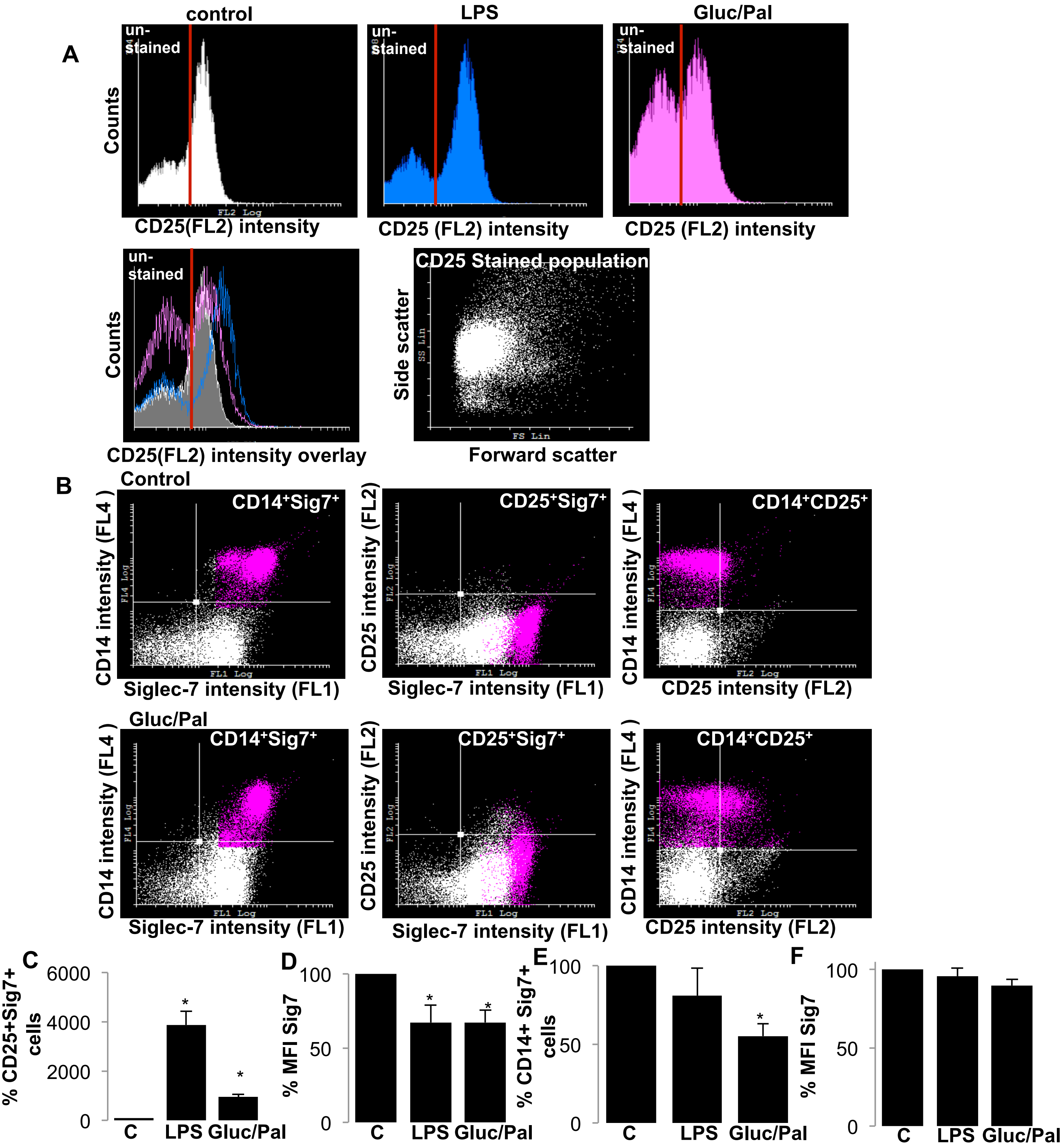

Supplementary Figure 3: FACS analyses in monocytes. (A) Cell surface expression of CD25 in elevated glucose and palmitate treated PBMCs (n=6) after 12h was determined using flow cytometry. Histograms for intensity of CD25 (FL2 filter) were plotted and overlayed to observe the effect of these treatments. *p<0.05 to 11.1 mM glucose treated monocyte fraction.

(B) PBMCs purified from buffy coats of blood donors (n=6) were treated with elevated glucose and palmitate for 12h. They were triple stained for Siglec-7, CD14 and CD25 and analyzed using flow cytometry. Their intensities were plotted against each other, and the quadrants were analyzed for number of positive cell and signal intensities, **(B,C)** the % cells co-labeled for CD25 and Siglec-7 quantified, and **(D)** the % mean fluorescent intensities of Siglec-7 in these cells plotted. **(B,E)** % cells co-labeled for CD14 and Siglec-7 were quantified, and the **(F)** % mean fluorescent intensities of Siglec-7 in these cells plotted. *p<0.05 to 11.1 mM glucose treated monocyte fraction.

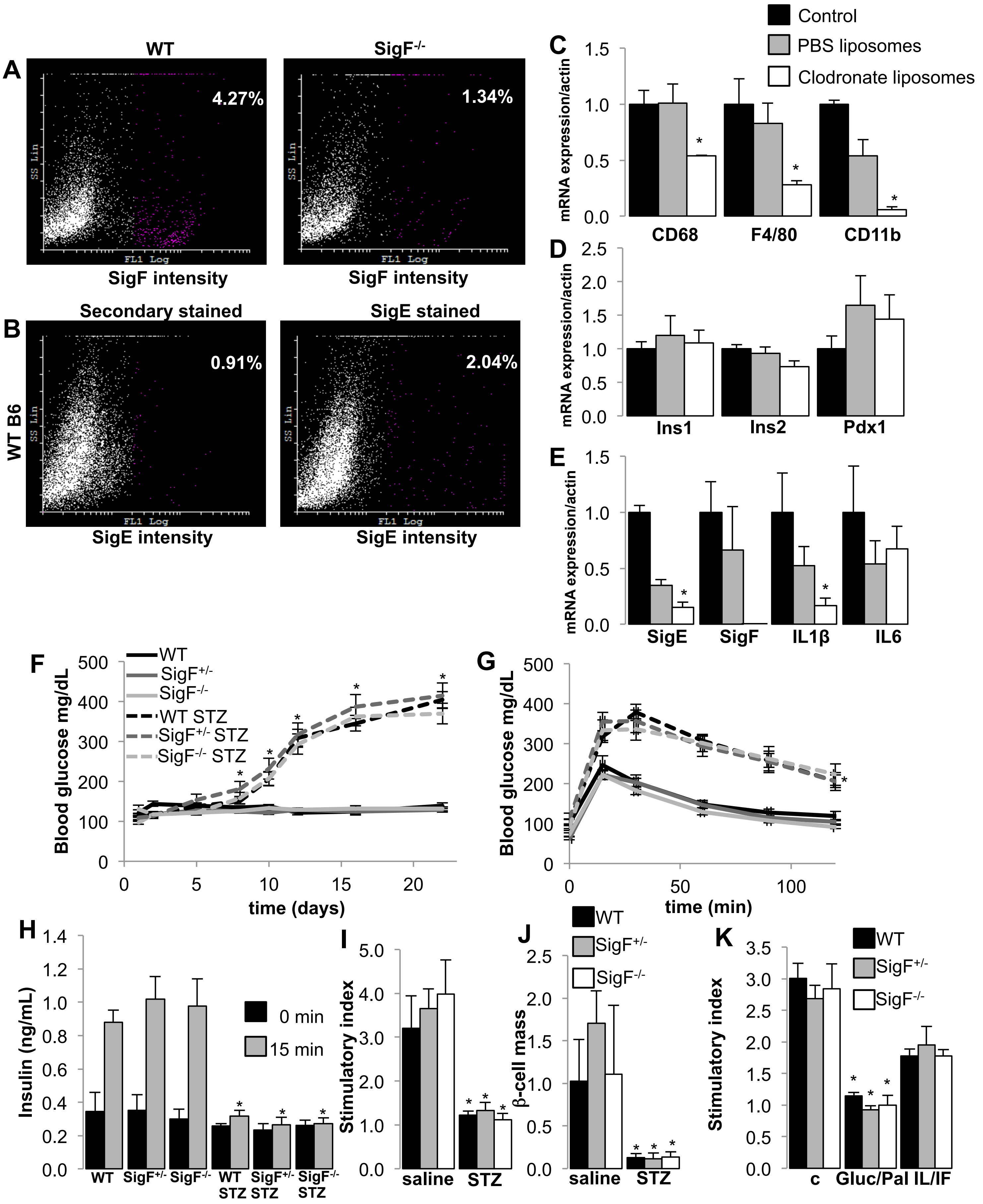

Suppl.Figure 4

Supplementary Figure 4: Siglec functional paralogs are absent in mouse endocrine cells.
Isolated mouse islets from C57Bl/6 WT and Siglec-F$^{-/-}$ were dispersed by Accutase treatment till a single cell state and plated on extracellular matrix-coated dishes. After one day of recovery time cells were scraped off, fixed and stained for FACS analysis. Data show one representative of three experiments; numbers indicate the percentage of stained cells **(A, B)**. Mouse islets were isolated, cultured in suspension and then treated with clodronate (Clo) or PBS containing liposomes (PBS) for 48h. RNA was isolated, reverse transcribed and analyzed for the indicated genes by qRT-PCR **(C-E)**. Eight-week-old male Siglec-F$^{-/-}$ mice and their littermate wildtype (WT) and heterozygous (Siglec-F$^{+/-}$) controls where injected with 50 mg/kg streptozotocin (STZ) or citric buffer (control) on 5 consecutive days (n=12-15). Random blood glucose measurements **(F)**, glucose tolerance tests **(G)** and *in vivo* glucose stimulated insulin secretion **(H, I)** were performed after 20 days. Stimulatory index show the amount of glucose stimulated divided by the amount of basal insulin secretion. **(J)** Mice were sacrificed at day 21. β-cell mass was analyzed from all treatment groups from 3 mice from 10 pancreatic sections/ mouse spanning the whole pancreas. **(K)** Isolated mouse islets of all three genotypes were plated on extracellular matrix-coated dishes and exposed to 22.2 mM glucose with palmitate (0.5 mM) or the cytokine mixture IL-1β (2 ng/ml) and IFNγ (1,000 U/ml) for 72h. Insulin stimulatory index denotes the ratio of secreted insulin during 1-h incubation with 16.7 mM glucose to that secreted during 1-h incubation with 2.8 mM glucose **(J)**. All data are means +/-SE from at least 15 mice/group in 3 independent experiments, *p<0.05 to control.

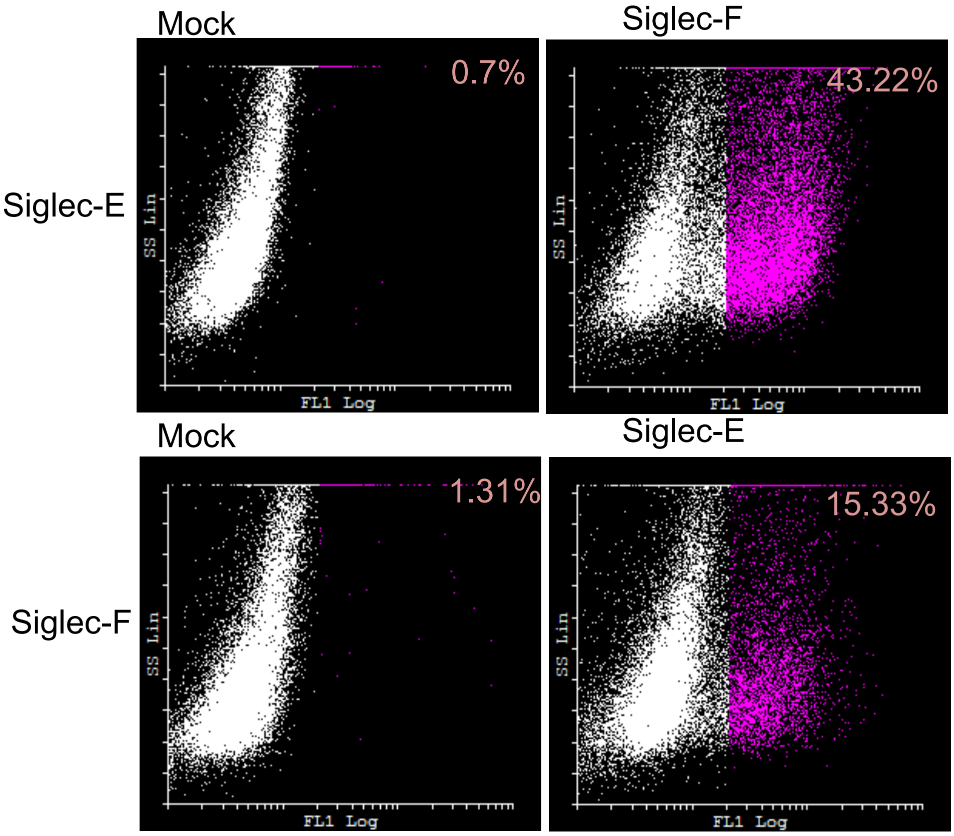

Supplementary Figure 5: **Evaluation of Siglec-E,-F antibodies.** HEK293T cells were transfected with LacZ, Siglec-E or Siglec-F plasmids, stained for Siglec-E or –F, respectively and analyzed using flow cytometry. Histograms indicate the intensity of FL1 for Siglec-E/-F stained cells.

Siglec-7 restores β-cell function and survival and reduces inflammation in pancreatic islets from patients with diabetes

Gitanjali Dharmadhikari *et al.*

Full Blots

Figure 4 C

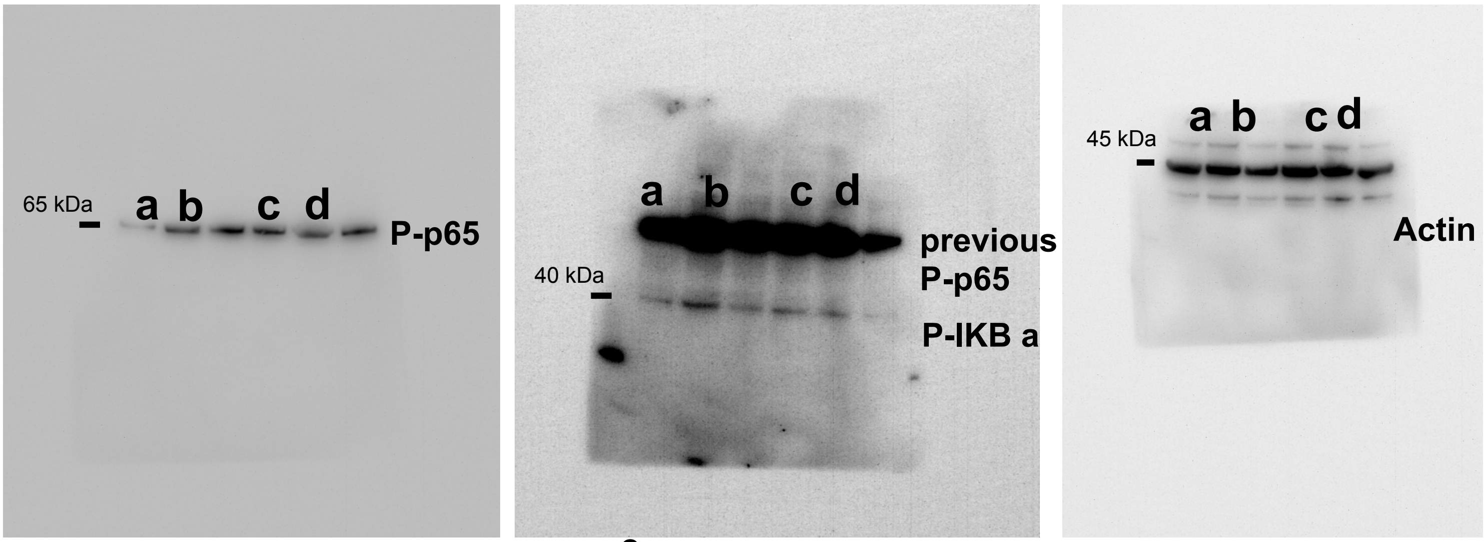

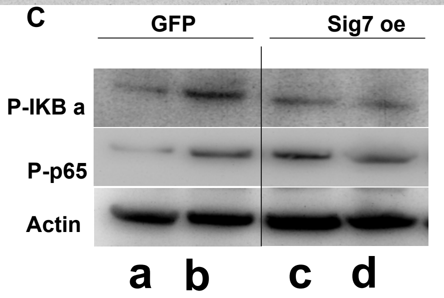

Figure 4 D

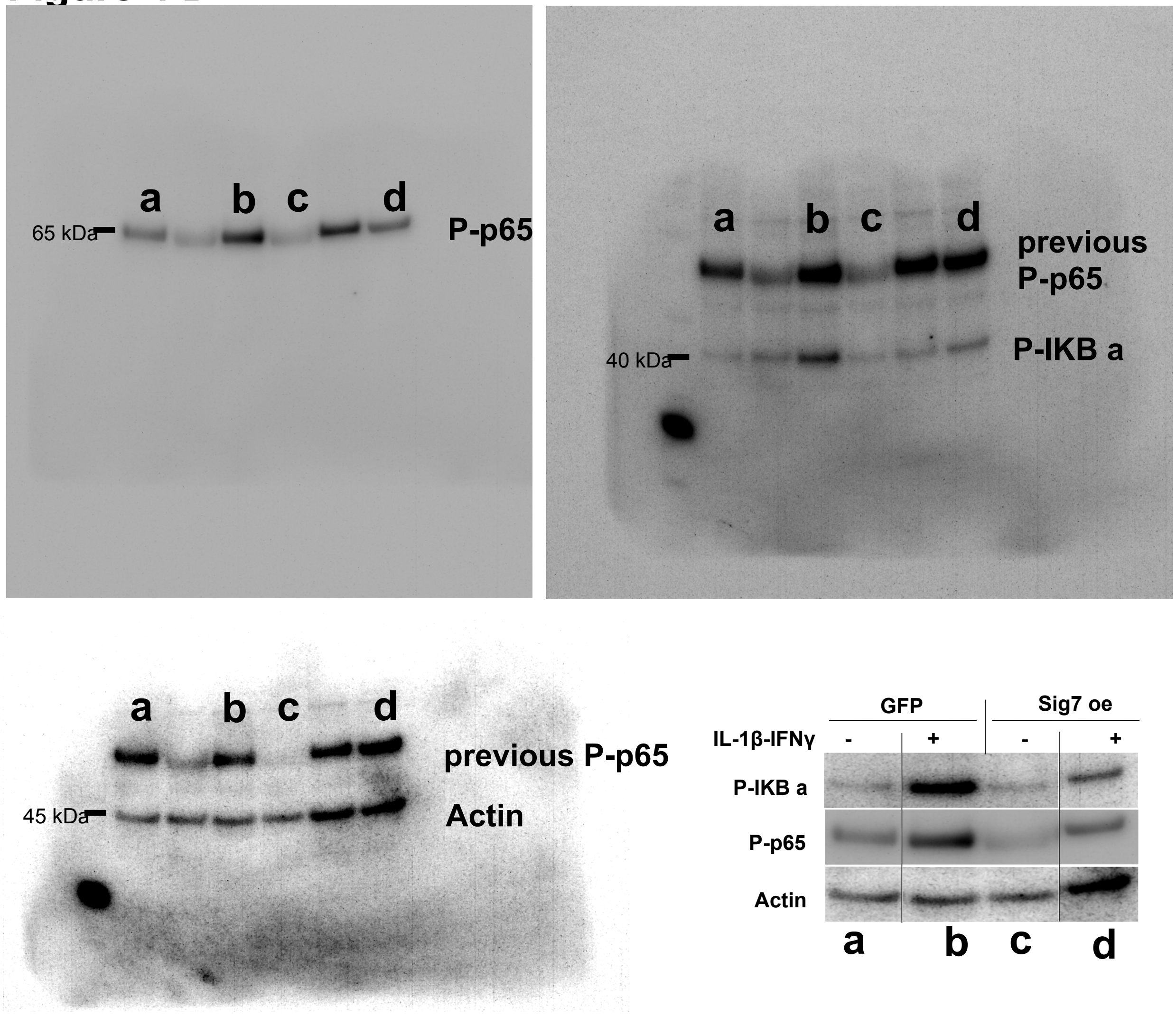

Figure 4 H

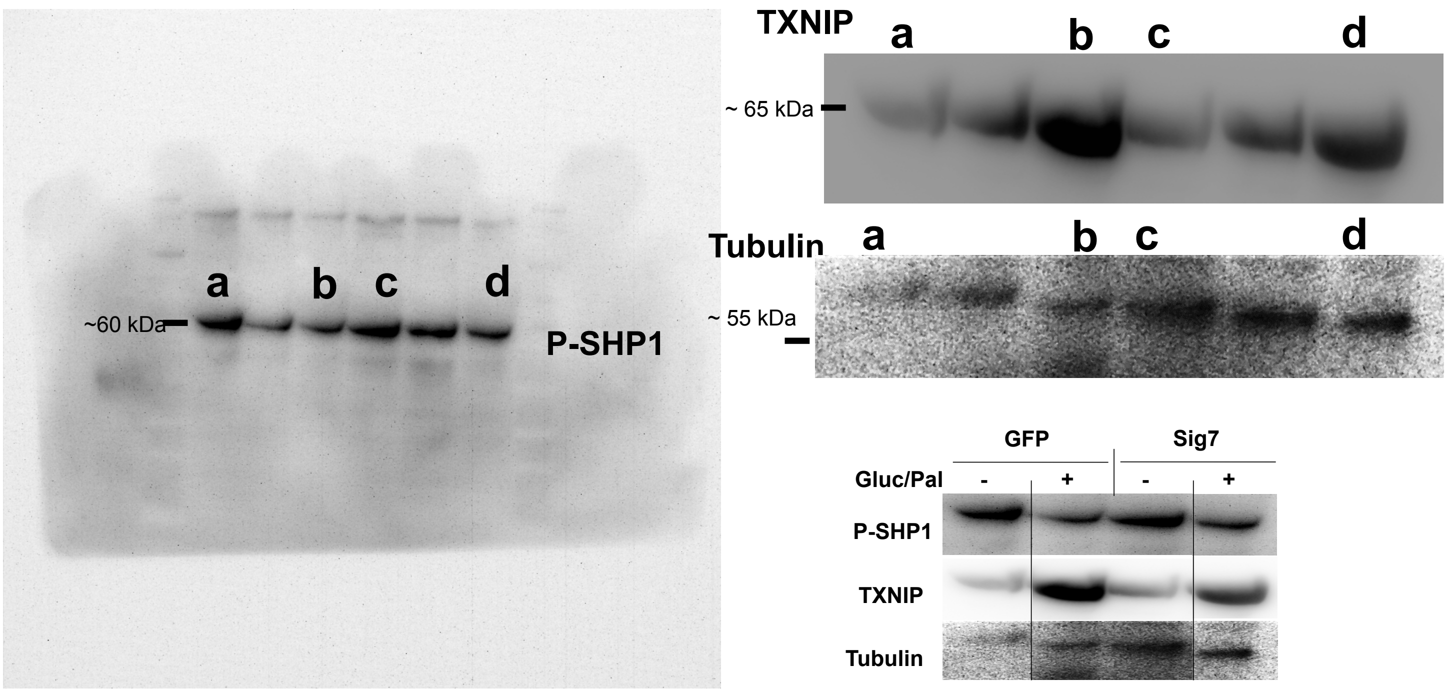

Figure 4 J

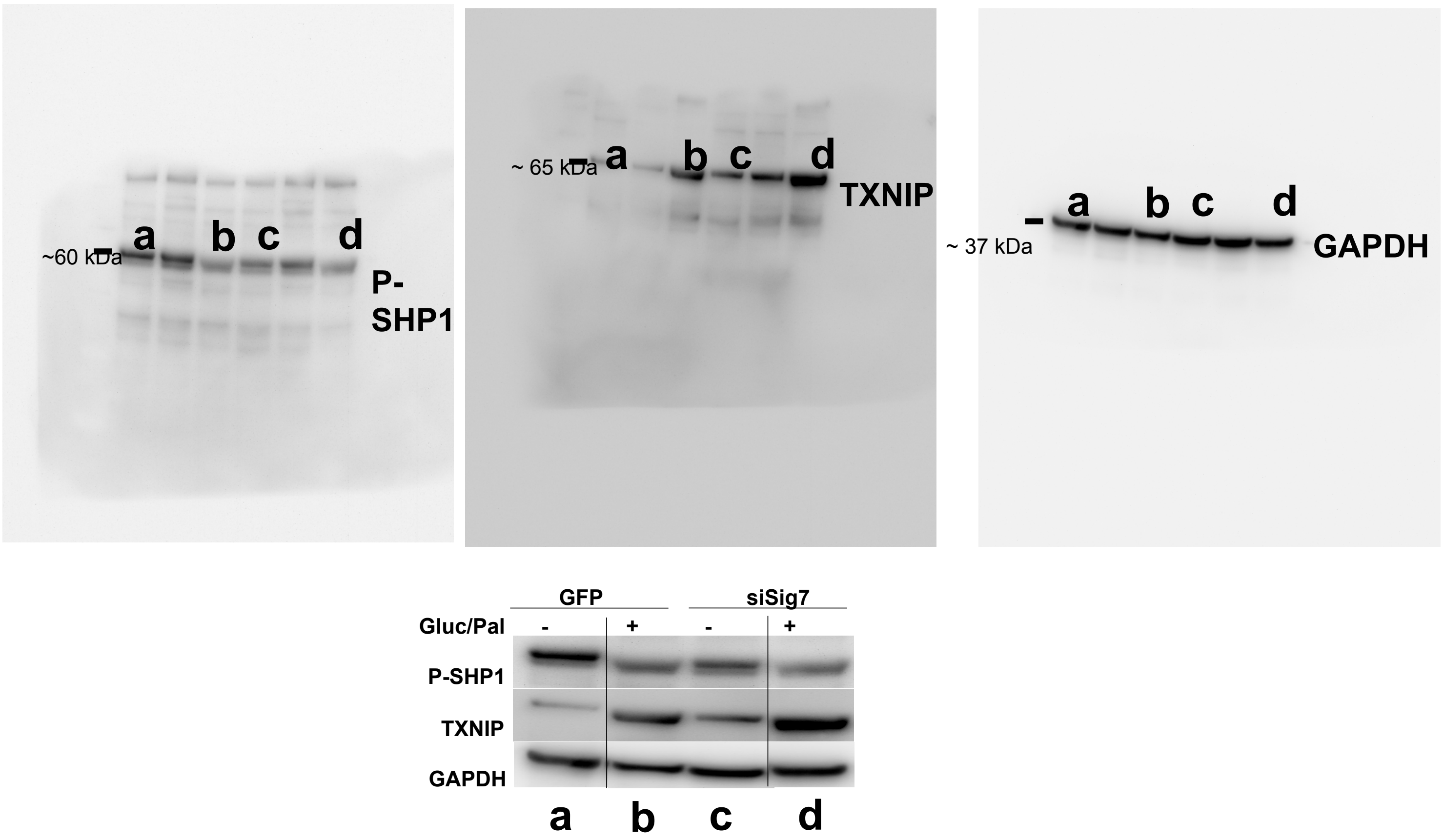

Suppl.Fig.1E

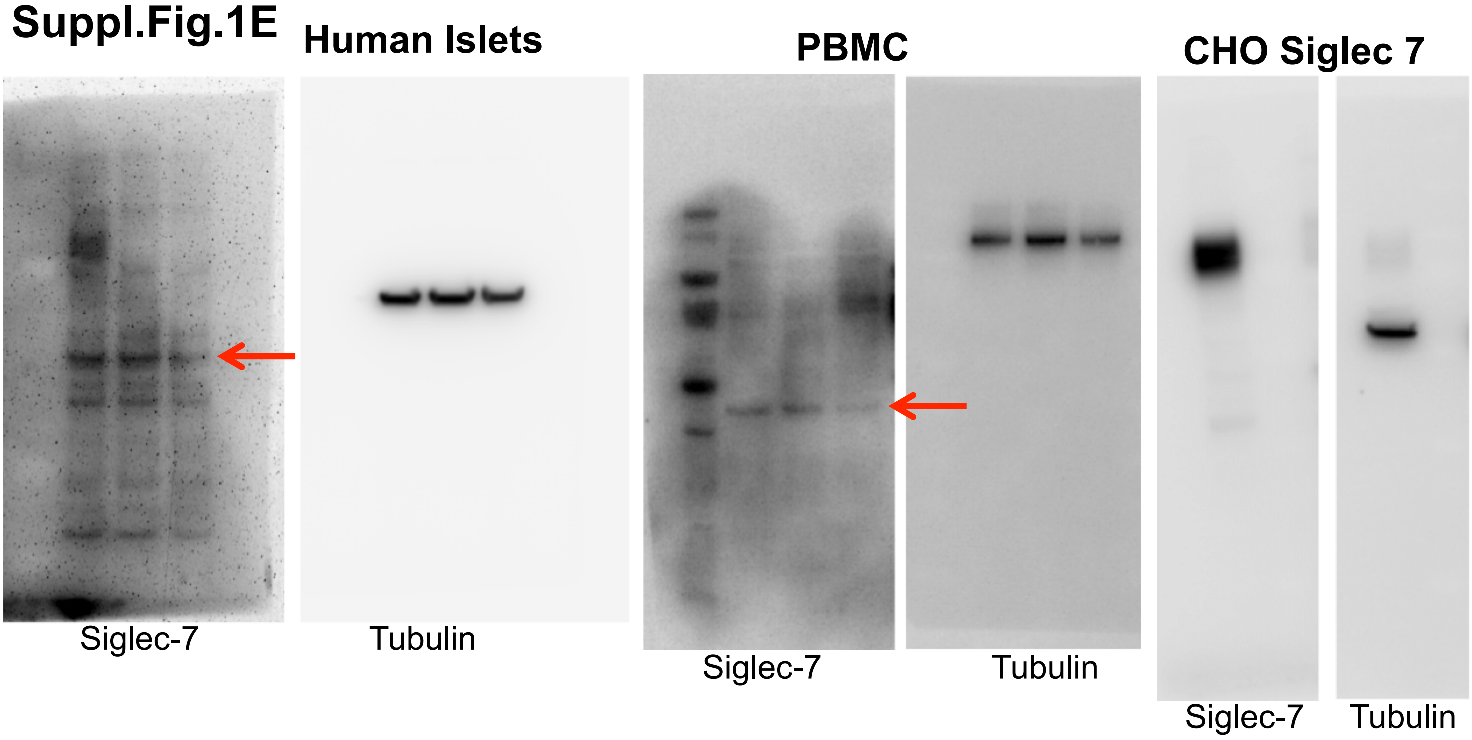

E

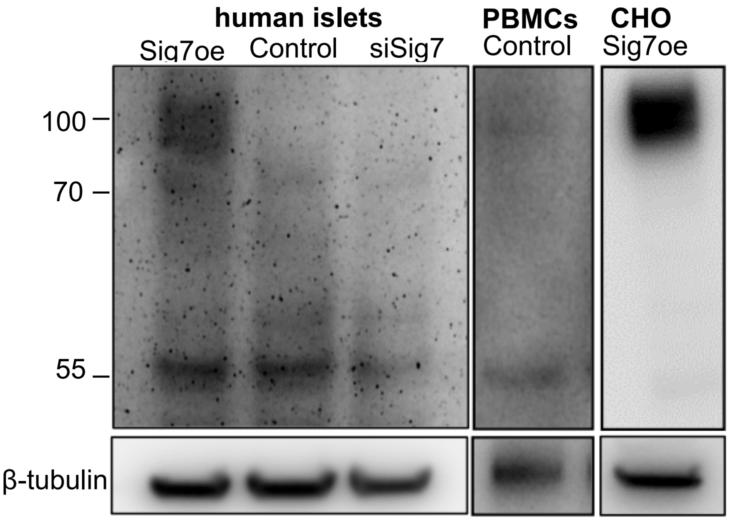

Supplement: Supplementary Dataset [file srep45319-s1.pdf]
